# Determining causes of death through an abridged verbal autopsy tool: a pilot test conducted among vulnerable communities in Kolkata, India

**DOI:** 10.1136/bmjgh-2025-022829

**Published:** 2026-07-20

**Authors:** Suman Kanungo, Melissa Glenda Lewis, Diplina Barman, Prantick Kumar Bhunia, Suparna Chatterjee, Sandip Samanta, Dipti Yadav, Sudipta Pati, Rounik Talukdar, Shubarna Chakraborty, Indrani Das, Amitabha Chattopadhyaya, Soumitra Ghosh, Kalpana Datta, Henry D Kalter, Debarati Guha-Sapir

**Affiliations:** 1 ICMR National Institute for Research in Bacterial Infections (ICMR-NIRBI), Kolkata, West Bengal, India; 2 Calcutta Medical College, Kolkata, West Bengal, India; 3 Institute of Postgraduate Medical Education and Research, Kolkata, West Bengal, India; 4 Dr B C Roy Post Graduate Institute of Pediatric Sciences, Kolkata, West Bengal, India; 5 Infectious Diseases & Beleghata General Hospital, Kolkata, West Bengal, India; 6 Johns Hopkins Bloomberg School of Public Health, Baltimore, Maryland, USA; 7 Centre for Research on the Epidemiology of Disasters, Universite Catholique de Louvain - Ecole Sante Public, Louvain, Belgium

**Keywords:** Decision Making, Global Health, Child health, Epidemiology, Public Health

## Abstract

**Background:**

Users often regard the 2022 WHO verbal autopsy (VA) instrument as lengthy and time-consuming to implement, emphasising the need for a more concise and practical tool, particularly for use in unstable or resource-limited settings. This study aimed to develop, pilot test and validate an abridged version of the WHO VA instrument capable of identifying probable causes of neonatal (0–27 days), child (1 month–11 years) and adult (12–49 years) deaths in vulnerable populations.

**Methods:**

An abridged VA instrument based on the expert algorithm method was developed and applied to deaths recorded between 2021 and 2022 in three tertiary general hospitals and one tertiary paediatric hospital in Kolkata, West Bengal. VA interviews were conducted between July 2024 and January 2025. Causes of death were assigned using two approaches: (1) underlying medical record diagnoses and (2) a hierarchical computer-based VA algorithm. Diagnostic metrics were used to validate algorithmic causes against medical diagnoses.

**Results:**

Among 270 deaths (90 per age group), 95.5% yielded a VA diagnosis. Among neonates, the leading causes were sepsis (34.4%: 83.8% VA sensitivity, 94.9% VA specificity), IPRE (25.6%: 69.6%, 97.0%) and pneumonia (16.7%: 100%, 98.7%). Among children, the two most common medical diagnoses were pneumonia (53.3%: 100%, 50%) and other infections including sepsis without a known source (25.6%: 21.7%, 98.5%). Among adults, the two most common medical diagnostic categories were acute febrile illness (38.5%: 80.9%, 98.6%) and cardiovascular diseases (38.5%: 100%, 98.6%). The accuracy of the new VA instrument compared with the medical diagnosis was 81.1% for neonates, 71.1% for children and 87.8% for adults, with an overall accuracy of 80%.

**Conclusion:**

The abridged VA instrument demonstrated satisfactory accuracy in identifying the probable causes of death across all age groups in the study populations. This instrument holds potential for enhancing cause-of-death reporting in resource-limited settings.

**WHAT IS ALREADY KNOWN ON THIS TOPIC**

- Verbal autopsy (VA) remains a vital approach for identifying the causes of death in settings where medical certification is often unavailable. The 2022 WHO VA instrument has been widely promoted for this purpose but is frequently considered too lengthy and difficult to administer, particularly in low-resource or high-risk environments.

**WHAT THIS STUDY ADDS**

- This study presents a newly developed abridged VA instrument consisting of 54–61 or fewer questions per age group (neonates, children and adults), compared with hundreds of questions in the 2022 WHO VA questionnaire. The results showed strong agreement with clinical diagnoses, particularly for neonatal sepsis, childhood infections and adult cardiovascular diseases. The tool demonstrated high diagnostic accuracy across all age groups, supporting its suitability for use in time-limited and resource-limited field settings.

**HOW THIS STUDY MIGHT AFFECT RESEARCH, PRACTICE OR POLICY**

- The simplified VA tool offers a practical, efficient alternative for mortality surveillance where resources are limited. Its integration into public health programmes could enhance the speed and quality of death reporting and strengthen evidence-based planning in health systems lacking complete death certification.

## Introduction

 Planning for public health, evaluating programmes and tracking progress toward global health goals, such as the Sustainable Development Goals, all depend on timely and accurate mortality data.^1^ However, civil registration and vital statistics (CRVS) systems remain weak in many low- and middle-income countries (LMICs), particularly in resource-limited settings. As a result, many deaths, particularly those that take place outside of medical facilities, go unreported or have no medically verified cause, undermining national and international health surveillance systems.^2–4^ This is especially and crucially true among populations who are living in disaster or conflict affected conditions, who number in the millions. Most are some of the poorest in the world and many live in these conditions for years. Little is known about the mortality patterns in these communities and outbreaks of emerging and re-emerging diseases have been observed to have initiated in these settings. Appropriate tools and methods to ascertain causes of deaths in such contexts do not exist and mortality patterns remain obscure.^5^

In 2019, of 55 million global deaths, 70% were registered, but only 42% had a medically verified cause of death (COD).^6^ In India, while the Civil Registration System registered 92% of the deaths that occurred in 2019, only 22.5% had a medically certified COD.^7 8^ In the state of West Bengal, only 73% of the deaths expected in 2017 were registered and a COD was certified for only 13% of these deaths.^9^ These discrepancies underscore the gap between formal death registration and medically meaningful documentation of mortality causes.

COD patterns are early and central indicators of impending health crises. By the time suspicion of any disease pathogen is confirmed by laboratory tests, it is almost always too late to mount an effective containment response.^10^ Lately, this gap in death monitoring has been recognised following Ebola, measles and, more recently, the Marburg outbreak in insecure zones. Even when there is a rudimentary death record, COD is often registered in vague terms such as cardiac or respiratory arrest, rendering these records of little use in disease containment or planning.

To address this gap in COD ascertainment, especially in communities without access to medical certification, the verbal autopsy (VA) method has been developed and increasingly adopted.^11–13^ VA is a structured interview conducted with the relatives or caregivers of the deceased to elicit observable signs and symptoms that occur prior to death.^14^ These symptoms are typically recognisable by laypersons and are used to infer the likely biological COD. Interpretation of VA data may be carried out by trained physicians or, more recently, through automated computer algorithms such as InterVA, SmartVA (tariff method) and InSilicoVA, all of which apply probabilistic techniques^12^ and the deterministic expert algorithm (EAVA) method.^15^ These tools have significantly enhanced the consistency, scalability and cost-efficiency of VA implementation, particularly in settings where physician review is impractical.

The most recent version of the WHO standardised VA instrument, released in 2022, reflects these priorities, although its widespread adoption has been delayed due to ongoing development and testing of the supporting probbase data and software. Development of the standardised VA instrument has been led by successful global collaborations, including the WHO, the Institute for Health Metrics and Evaluation and the Bloomberg Philanthropies Data for Health Initiative.^16^ These efforts have emphasised methodological consistency, cross-country comparability and integration into national CRVS and mortality surveillance systems.^17^ However, despite its methodological rigour, the 2022 WHO VA instrument has been criticised for its length and complexity. Interviews often exceed 45 min, leading to interviewer fatigue, reduced data accuracy and low respondent compliance.^14 18 19^ These challenges are especially acute in environments where data must be collected rapidly and at scale, such as during humanitarian emergencies, in remote areas or within overstretched health systems. Studies have shown that lengthy VA instruments limit routine implementation due to high training costs and respondent fatigue. This has made it harder to use VA in real-time monitoring systems and reduced its usefulness for quickly responding to health emergencies. Field practitioners have repeatedly emphasised the need for shorter, more operationally feasible VA tools that retain diagnostic validity while reducing the burden on both interviewers and respondents.^20–22^

To address these operational barriers, the present study aims to develop, pilot test and validate an abridged version of the WHO VA instrument. Prior to testing an abridged prototype in crises settings, which are often insecure and difficult to access, we decided to first design and test a version in a resource-constrained setting with communities that are like those in humanitarian contexts. This shorter tool is designed to capture probable causes of neonatal, child and adult deaths with sufficient diagnostic precision, while being better suited for use in vulnerable populations and resource-constrained settings. The goal is to strengthen the quality and timeliness of cause-of-death data, thus enabling more responsive, equitable and data-driven public health action.

## Methodology

### Study design and participants

A record-based observational study was conducted among deceased neonates (0–27 days), children (1 month–11 years) and adults (12–49 years) for validation of the abridged VA tool. Death records were collected over 2021–2022 from three state-run tertiary care general hospitals and one tertiary paediatric hospital located in Kolkata, West Bengal.

### Sample size and sampling technique

The sample size was desired to enable estimation of 75% VA sensitivity with 95% confidence, ±10% precision and 10% non-response rate. Practical considerations limited sample size to 90 decedents in each age group (online supplemental figure S1). For neonates, children and adults, respectively, this required assuming cause-specific mortality proportion (CSMP) of 50%, 50% and 30% for the most common cause. A list of deaths of each age group with a medical COD and residence within 50 km of the institute was generated, and a random sample of deaths was selected from each list for the VA interview.

### Abridged VA instrument

An abridged instrument developed for this study, designed to identify COD using the EAVA analysis method, consists of three age-specific versions tailored for neonates, children and adults, comprising 61, 60 and 54 closed-ended questions, respectively. These were derived from the 2022 WHO VA instrument by prioritising essential questions relevant to the most common causes of death in each age group while eliminating redundancies and less informative items. The structure of the tool preserves critical domains such as signs and symptoms preceding death, duration of illness and relevant medical history, allowing for reliable cause-of-death determination. Most of the neonatal and child questions included in the abridged instrument were based on algorithms developed by researchers for prior VA validation studies and a literature review to identify illness signs and symptoms commonly associated with particular neonatal and child illnesses.^15^ Newer algorithms, developed for neonatal jaundice, neonatal haemorrhagic syndrome, AIDS and haemorrhagic fever, and the pertussis algorithm, adapted from the US Centers for Disease Control and Prevention case definition were included in a validation study of the EAVA hierarchies used for the current study.^23 24^ The adult questions and hierarchies used by the current study were adapted from Chandramohan *et al*’s previously developed and validated instrument.^20^ The abridged questionnaire was piloted among 15 deaths (five in each age group), and minor adjustments were made prior to field implementation (online supplemental appendix 1–3)

### Data collection methods

The designated study physician collected the information (inclusive of demographics, diagnoses and underlying COD) from the medical records department of the study hospitals. The detailed data were entered in MS Excel (V.21), and a unique identification number was assigned to each case for linkage with the VA data. VA data collection was carried out by a team of experienced field workers, each holding a bachelor’s degree and having over 10 years of experience in health-related field data collection. The team had a strong background in community-based interviews and demonstrated familiarity with sensitive data collection practices. Prior to the start of the study, all team members underwent a comprehensive 3-day training session conducted by SK and DG-S. The training included detailed instructions on the use of the VA instrument, interview techniques, ethical considerations and mock interview sessions. These mock sessions were closely supervised for consistency and clarity.

Following the training, the team visited the selected households to conduct VA interviews. The primary caregiver of the deceased, who was considered the most reliable respondent, was approached. Prior appointments were scheduled before each interview. Interviews were conducted one-on-one in a private, well-lit space within the respondent’s home. Before beginning the interview, the interviewer conducted informed consent and spent time building rapport, explaining the purpose of the study and allowing the respondent to ask questions, helping ensure the respondent was emotionally and physically comfortable. The interviews were audiorecorded and notes were taken in the local languages (Bengali and Hindi). Transcripts were later translated into English by SP and DY and verified through follow-up telephonic confirmation with the respondents. The data were entered into MS Excel (V.21) and quality control procedures were followed to ensure accuracy. On average, each VA interview lasted approximately 30–35 min. In comparison, studies using the 2022 WHO VA report interview durations exceeding 45–60 min depending on the type of setting and implementation.^18 19^ Data collection took place from October to December 2024.

### Methodology for assigning VA causes of death

For adult deaths, we adapted the hierarchical, expert-opinion-based validated algorithms described by Chandramohan *et al* to assign the VA underlying COD and categorised the causes according to the classifications provided in the same study.^20^ For neonatal and child deaths, we used the algorithms and hierarchies developed by Kalter *et al* which follow a structured approach to determining and classifying the underlying COD.^15 24^ For each death, a software programme reviewed the responses to all VA questions to determine which cause(s) met the algorithm’s criteria. These were then passed through a hierarchy to assign a single main COD. This process was implemented using STATA V.16. Output of the VA analysis consists of the CSMP, also referred to as cause-specific mortality fraction, for each age group, which is the proportion of all deaths in a group due to each cause that the VA examines for that group.

### Validation of the VA causes of death

Validation of the VA causes of death for each age group was conducted by comparing the VA causes for all deaths to the medical causes of the same deaths, with the medical causes serving as the reference standard against which the VA findings were judged. The VA’s validity for each individual COD was assessed using several measures, including sensitivity, specificity, positive predictive value (PPV), negative predictive value (NPV) and Youden’s Index. Sensitivity was defined as the proportion of VA positives compared with all deaths of the same age group with the same medical cause, while specificity represented the proportion of VA negatives compared with all deaths of the same age group without the same medical cause. PPV was defined as the proportion of VA true positives divided by VA true positives plus VA false positives, while the NPV was defined as the proportion of VA true negatives divided by VA false negatives plus VA true negatives, and a Youden’s Index value >0.7 was considered indicative of good diagnostic performance.^25^ The overall accuracy of the VA CSMP of all causes together was compared with the CSMP of the medical causes, which is measured from 0 to 1 and is interpretable as percentage accuracy.^26^ All the analyses were performed in STATA V.16.^27^

## Patient and public involvement

- When and how were patients/public first involved in the research?

Patients were not involved in the design or planning of the research. Bereaved family members were involved only as respondents during VA interviews conducted after the study tools had been developed.

- How were the research question(s) developed and informed by their priorities, experience and preferences?

The research question was developed based on existing evidence and field experience indicating that the standard WHO VA instrument is lengthy and operationally burdensome in resource-limited settings. It was not directly informed by patient or public input.

- How were patients/public involved in:

- Design and conduct of the study?

Patients and family members were not involved in the design or conduct of the study. Their involvement was limited to participation as respondents in VA interviews.

- Choice of outcome measures?

No involvement.

- Recruitment to the study?

Not applicable.

- How were (or will) patients/public be involved in choosing the methods and agreeing on dissemination?

Not applicable.

## Ethical clearance

The study received approval from the institutional ethics committee of the ICMR National Institute of Cholera and Enteric Diseases, Kolkata (IEC approval No A-10 (2)/IEC-2023) dated August 2023. Written informed consent was obtained from all adult respondents participating in the VA interviews. For deaths among children and neonates, consent was provided by the adult family member or legal guardian who served as the VA respondent. The study was also approved by the Health Ministry Screening Committee dated 09 November 2023, Ministry of Health and Family Welfare, Government of West Bengal, India (Letter No. HFW-27039/-CD SEC dept. of 4H&FW/239, dated 23 November 2023) for accessing the data from medical record sections of the hospitals. The study is registered in Clinical Trial Registration of India (https://www.ctri.nic.in).

## Funding

This study is partially funded by the project Societies at Risk, Uppsala University Sweden through its grant from Riksbankens Jubileumsfond.

## Results

### Respondents’ characteristics

A total of 270 respondents, one per decedent, were included in the study. Most respondents for neonatal and child deaths were parents (mother/father), whereas for adult deaths, relationships to the deceased were more varied (table 1). Most respondents reported living with the deceased at the time of death and were aged early to late 30s. In terms of educational attainment, a majority had at least completed middle school across all groups.

**Table 1:** Characteristics of the respondents in the study

		Neonates (n=90)	Child (n=90)	Adult (n=90)
Variables	Categories	n (%)	n (%)	n (%)
Relationship with deceased	Wife/husband	–	–	31 (34.4)
	Mother	32 (35.6)	43 (47.8)	–
	Father	45 (50.0)	38 (42.2)	–
	Mother/father	77 (85.6)	81 (90.0)	13 (14.4)
	Brother/sister	–	–	18 (20.0)
	Son/daughter	–	–	16 (17.8)
	Grandfather/grandmother	6 (6.7)	4 (4.4)	–
	Brother-in-law/sister-in-law	–	–	6 (6.7)
	Another close relative	7 (7.8)	5 (5.6)	6 (6.7)
Lived with the deceased	Yes	83 (92.2)	88 (97.8)	89 (98.9)
Sex	Male	57 (63.3)	46 (51.1)	57 (63.3)
	Female	33 (36.7)	44 (48.9)	33 (36.7)
Age in years	Median (IQR)	31 (27–36)	32 (26–37)	38 (29–48)
Highest standard of education	Illiterate and literate with no formal education	7 (7.8)	6 (6.7)	5 (5.6)
	Literate, primary or below	12 (13.3)	11 (12.2)	13 (14.4)
	Secondary (middle to class XII)	58 (64.5)	62 (68.9)	59 (65.6)
	Graduate and above	13 (14.4)	11 (12.2)	13 (14.4)

## Deceased characteristics

Among the deceased, males accounted for just over half in all age groups. On average, neonates and children were on the younger side of their age groups, while adults tended towards the older. Most children were too young to have attended school, while more than 70% of adults had achieved a secondary or higher education level and more than half were employed (table 2).

**Table 2:** Characteristics of the deceased individuals in the study

Variables	Categories	Neonates (n=90)	Child (n=90)	Adult (n=90)
		N (%)	N (%)	N (%)
Sex	Male	51 (56.7)	51 (56.7)	46 (51.1)
	Female	39 (43.3)	39 (43.3)	44 (48.9)
Age	0–6 days	47 (52.2)	–	–
	7–27 days	43 (47.8)	–	–
	Median days (IQR)	6 (2–11)	–	–
	1–23 months	–	55 (61.1)	–
	2–4 years	–	16 (17.8)	–
	5–11 years	–	19 (21.2)	–
	Median years (IQR)	–	0.83 (0.3–3.5)	–
	12–19 years	–	–	4 (5.1)
	20–34 years	–	–	21 (26.9)
	35–49 years	–	–	65 (72.2)
	Median years (IQR)	–	–	39 (34–47)
Highest standard of education	Illiterate and literate with no formal education	–	79 (87.8)	13 (14.4)
	Literate, primary or below	–	10 (11.1)	13 (14.4)
	Secondary (middle to class XII)	–	1 (1.0)	53 (58.9)
	Graduate and above	–	–	11 (12.2)
Occupation	Non-worker	–	–	40 (44.4)
	Wage earner (daily wage/informal labour)	–	–	29 (32.2)
	Salaried (formal employment)	–	–	8 (8.9)
	Cultivator/farmer	–	–	4 (4.4)
	Profession/business	–	–	6 (6.7)
	Student	–	–	3 (3.3)

### Neonatal causes of death and VA algorithm performance

Sepsis was the leading cause of neonatal death, accounting for approximately one-third of hospital deaths, followed by intrapartum-related events (IPRE) and pneumonia (table 3). The VA algorithm showed high sensitivity and specificity for sepsis and pneumonia, while diagnostic performance for IPRE was moderate. Approximately one in six neonates died from a prematurity-related cause, for which VA had moderate sensitivity and high specificity. A small proportion of the neonates died from haemorrhagic syndrome, congenital malformation and neonatal jaundice; these causes had lower PPVs despite moderate to high sensitivity. The VA cause for 3.3% of deaths remained unspecified. Youden’s Index indicated strong diagnostic performance for several of these causes, with lower discriminative ability noted for congenital malformations. The overall CSMP accuracy of the VA algorithms compared with the medical diagnoses was 81.1%.

**Table 3:** Summary statistics for neonatal causes of death

Causes of death	Algorithm n (%)	Hospital n (%)	Sensitivity (%)	Specificity (%)	PPV (%)	NPV (%)	Youden’s Index
Sepsis	29 (32.2)	31 (34.4)	83.8	94.9	89.7	91.8	0.79
IPRE*	18 (20.0)	23 (25.6)	69.6	97.0	88.9	90.2	0.66
Pneumonia	16 (17.8)	15 (16.7)	100	98.7	93.7	100	0.99
Prematurity	13 (14.4)	14 (15.6)	78.6	97.4	84.6	96.1	0.76
Haemorrhagic syndrome	4 (4.4)	2 (2.2)	100	97.7	50.0	100	0.99
Congenital malformation	4 (4.4)	3 (3.3)	66.7	97.7	50.0	98.8	0.64
Neonatal jaundice	3 (3.3)	1 (1.1)	100	97.8	33.3	100	0.98
Others†	0 (0.0)	1 (1.1)	0	100	–	98.9	0
Unspecified	3 (3.3)	0 (0.0)	–	96.7	0	100	*Undefined*
**Total (N**)	**90**	**90**					

* *IPRE includes birth asphyxia and birth trauma*.

† *Others: intestinal perforation associated with Hirschsprung disease.*

IPRE, intrapartum related events; NPV, negative predictive value; PPV, positive predictive value.

### Child causes of death and VA algorithm performance

Pneumonia and other infections together accounted for nearly 80% of hospital diagnosed child CODs (table 4). Sensitivity and specificity, respectively, for these two causes were very high. However, the inclusion of possible pneumonia/acute lower respiratory infection (ALRI) in the VA algorithm for pneumonia, and sepsis without a known source as one of the medically diagnosed other infections, resulted, respectively, in low specificity and sensitivity and corresponding low diagnostic accuracy (Youden’s Index) for these causes. Other communicable diseases caused 10% of the child deaths, while injury and malnutrition were responsible for about 3% of the deaths. The VA algorithm had very good to excellent diagnostic accuracy for all these causes. Additionally, the algorithm lacked diagnostic code for the 7.8% of hospital diagnosed non-communicable diseases (NCD) and 3.3% of VA causes were unspecified. The overall CSMP accuracy of the VA algorithms compared with the medical diagnoses was 71.1%.

**Table 4:** Summary statistics for child causes of death

Cause of death	Algorithm n (%)	Hospital n (%)	Sensitivity (%)	Specificity (%)	PPV (%)	NPV (%)	Youden’s Index
Pneumonia*	69 (76.7)	48 (53.3)	100	50.0	69.6	100	0.5
Other infections†	6 (6.7)	23 (25.6)	21.7	98.5	83.3	78.6	0.20
Meningitis	5 (5.6)	6 (6.7)	83.3	100	100	98.2	0.83
Injury	2 (2.2)	2 (2.2)	100	100	100	100	1.00
Haemorrhagic fever	2 (2.2)	2 (2.2)	100	100	100	100	1.00
Malnutrition	2 (2.2)	1 (1.1)	100	98.8	50.0	100	0.99
Diarrhoea	1 (1.1)	1 (1.1)	100	100	100	100	1.00
Others‡	0 (0.0)	7 (7.8)	0	100	–	92.2	0
Unspecified	3 (3.3)	0 (0.0)	–	96.7	0	100	*Undefined*
**Total (N**)	**90**	**90**					

* VA pneumonia includes possible pneumonia or acute lower respiratory infection.

† ‘Other infections: 17 hospital causes were sepsis (five with a known source, 12 unknown), six were other conditions.

‡ *Others: cardiovascular disorders, hepatic failure, respiratory failure and leukaemia.*

NPV, negative predictive value; PPV, positive predictive value.

### Adult causes of death and VA algorithm performance

Adult causes were categorised into communicable diseases, direct maternal diseases and NCDs, with communicable and NCDs together accounting for nearly 93% of all deaths (table 5). Acute febrile illness (AFI) was a major contributor to communicable diseases, and the VA algorithm demonstrated high sensitivity and specificity for this cause category, as it did for individual causes within this category such as pneumonia and meningitis, and for other communicable diseases including rabies, diarrhoeal diseases and tuberculosis (TB)/AIDS. Other AFI (specified/unspecified) was less accurately captured, with a sensitivity of 27.3% and a PPV of 60%, suggesting partial misclassification. About 1.1% of adults had chickenpox encephalitis, as identified by hospital records, but this was missed by the VA algorithm due to coding limitations. The algorithm also made five false positive diagnoses of hepatitis.

**Table 5:** Summary statistics for adult causes of death

Cause of death	Algorithm n (%)	Hospital n (%)	Sensitivity (%)	Specificity (%)	PPV (%)	NPV (%)	Youden’s Index
I Communicable diseases	**34** (**37.8**)	**36** (**40.0**)	**91.7**	**98.2**	**97.1**	**94.6**	0.9
Acute febrile illness (AFI**)**	**18** (**20.0**)	**20** (**22.2**)	**80.9**	**98.6**	**94.4**	**94.4**	**0.8**
Pneumonia	7 (7.8)	7 (7.8)	100	100	100	100	1.0
Hepatitis	5 (5.6)	0 (0.0)	–	94.4	–	–	–
Meningitis/encephalitis	1 (1.1)	2 (2.2)	50	100	100	98.9	1.0
Other AFI (specified/unspecified)	5 (5.6)	11 (12.2)	27.3	97.5	60	90.6	0.25
Tuberculosis (TB) /AIDS	**4** (**4.4**)	**4** (**4.4**)	**100**	**100**	**100**	**100**	**1.0**
AIDS	1 (1.1)	1 (1.1)	100	100	100	100	1.0
Pulmonary TB	3 (3.3)	3 (3.3)	100	100	100	100	1.0
Diarrhoeal diseases	5 (5.6)	5 (5.6)	100	100	100	100	1.0
Rabies	7 (7.8)	7 (7.8)	100	100	100	100	1.0
II Direct maternal causes	**2** (**2.2**)	**2** (**2.2**)	**100**	**100**	**100**	**100**	**1.0**
Eclampsia	2 (2.2)	2 (2.2)	100	100	100	100	1.0
III Non-communicable diseases	**47** (**52.2**)	**51** (**56.7**)	**90.2**	**97.4**	**97.9**	**88.4**	**0.88**
Cardiovascular disorders	**21** (**23.3**)	**20** (**22.2**)	**100**	**98.6**	**95.2**	**100**	**0.99**
Congestive heart failure	7 (7.8)	7 (7.8)	100	100	100	100	1.0
Ischaemic heart disease	8 (8.9)	8 (8.9)	100	100	100	100	1.0
Cerebrovascular accident	6 (6.7)	5 (5.6)	100	98.8	83.3	100	0.99
Cirrhosis of liver	8 (8.9)	10 (11.1)	80	100	100	97.6	0.80
Acute abdominal conditions	4 (4.4)	4 (4.4)	100	100	100	100	1
Diabetes	1 (1.1)	1 (1.1)	100	100	100	100	1
Neoplasms	**8** (**8.9**)	**8** (**8.9**)	**75**	**98.8**	**85.7**	**97.6**	**0.74**
Gastrointestinal tract	5 (5.6)	4 (4.4)	100	98.8	80	100	0.99
Cervix/uterus	1 (1.1)	1 (1.1)	100	100	100	100	1
Breast	1 (1.1)	1 (1.1)	100	100	100	100	1
Lung	1 (1.1)	1 (1.1)	100	100	100	100	1
Liver	0 (0.0)	1 (1.1)	0	100	–	98.9	0
Renal disorders	5 (5.6)	4 (4.4)	100	98.8	80	100	0.99
Central nervous system disorder	0 (0.0)	3 (3.3)	0	100	–	96.7	0
All other specified non-communicable diseases (myxoedema coma)	0 (0.0)	1 (1.1)	0	100	–	98.9	0
IV Symptoms, signs and syndromes not elsewhere classified	**1** (**1.1**)	**1** (**1.1**)	**100**	**100**	**100**	**100**	**1**
Anaemia	1 (1.1)	1 (1.1)	100	100	100	100	1
V Unspecified	**6** (**6.7**)	0 (0.0)	–	93.3	-0	100	*Undefined*
Total (N)	**90**	**90**					

NPV, negative predictive value; PPV, positive predictive value.

Direct maternal deaths, all due to eclampsia, were rare but with full concordance between the VA algorithm and medical records. Cardiovascular disorders (CVD) emerged as the most common NCD category among adult deaths, with the algorithm demonstrating excellent performance for the three individual causes of congestive heart failure, ischaemic heart disease and stroke. Liver cirrhosis was the most common individual NCD among the hospital deaths and was detected with high sensitivity, while all cases of acute abdominal conditions and renal disorders, together accounting for nearly 9% of hospital deaths, were identified by VA. Neoplasms were a notable contributor to adult deaths with GI cancers being the most common, with just the one case of liver cancer not being detected by VA. Central nervous system conditions and other NCDs also went undetected due to coding limitations. Additionally, 6.7% of adult deaths remained unclassified by the algorithm. The overall CSMP accuracy of the VA algorithm compared with hospital diagnosis was 87.8%.

## Discussion

The importance of VA lies in its ability to reveal trends in causes of death in LMICs where the CRVS system is weak. This is particularly important in especially marginalised communities such as those composed of displaced persons or refugees, helping the public health sector identify major disease burdens,^28^ which along with appropriate reporting of deaths serves as the basis of any nation’s mortality surveillance system.^28 29^ Better understanding the CODs helps a nation plan multiple intervention measures and efficiently allocate resources towards the improvement of the population’s health. This is especially complex with adult COD patterns, which show a complicated interaction between external factors, non-communicable illnesses and communicable diseases.^28 29^ Increasing numbers of vertical programmes for the surveillance and prevention of communicable and NCDs in developing countries like India have called for more vigorous methods of identifying the CODs.^30^ Although several central registration services exist, there still remains a major gap in the identification of the primary CODs in the Indian population, making VA methods essential.^31^ All these factors are equally if not more central to providing more cost-effective services in refugee and displaced populations especially in the prevailing context of severe resource constraints. Existing VA tools are often long and complicated and impose a burden on resources for successful implementation.^32^ Hence, the present study was aimed at developing, pilot testing and validating an abridged version of the 2022 WHO VA instrument among neonates, children and adults.

For conducting a VA validation study, it is always important to have a valid reference diagnosis for comparison. In this study, the hospital death records were regarded as the ‘reference standard,’ and the VA tool-derived diagnosis was compared against it.^20^ The algorithms described by Chandramohan *et al* were applied for adult deaths, while those described by Kalter *et al* were used for neonatal and child deaths.^15 20^ Minor context-specific adaptations were made only for the adult algorithms, including combining AFI categories and introducing an additional decision rule for lung cancer (chronic cough >30 days + coughed up blood+no VA tuberculosis+noticeable weight loss). The VA questionnaire was designed to capture the information required to operationalise these algorithms.

The abridged VA instrument demonstrated moderate to high sensitivity and specificity for all leading causes of neonatal and child mortality except for low sensitivity and specificity, respectively, for childhood other infections and pneumonia, while achieving moderate (71%) to high (81%) overall CSMP accuracy. These values for neonates are comparable to an Indian hospital-based validation of the 2007 WHO VA tool, which reported overall accuracies ranging from 78% to 92% across different causes and to a study conducted among Pakistani neonates using the 2012 WHO VA tool, which reported sensitivities and specificities generally exceeding 75% for the leading causes of neonatal death.^33 34^

Pneumonia was the leading cause of child deaths in our study, identified in 53.3% of cases, consistent with findings from Murray *et al*, where 49% of child deaths were attributed to pneumonia by VA.^35^ The perfect sensitivity but low specificity and Youden’s Index of our VA algorithm for pneumonia, which compared poorly to Nichols *et al*’s reported VA diagnostic accuracy for pneumonia, appears to be due to our including code for ’possible pneumonia/ALRI’ as well as pneumonia, while the comparison hospital diagnosis included just pneumonia.^36^ The opposite situation explains our algorithm’s poor performance for childhood ‘other infections’. The 25.6% of deaths with this hospital assigned COD included 13.3% children who died from sepsis without a known source, which is difficult for VA to capture. These cases were likely classified under pneumonia or ALRI, reflecting overlapping clinical features. Similar limitations in distinguishing sepsis have also been noted by Leitao *et al.*^37^ Notably, the VA incorrectly attributed 5.6% of deaths to hepatitis, while hospital records confirmed these were actually due to dengue fever, which is similar to a study finding by Serina *et al.*^22^ This suggests that VA has difficulty in distinguishing hepatitis from dengue fever and that its performance might be improved by including hepatitis in the other AFI category. While acknowledging this limitation of VA in distinguishing some specific febrile illnesses, at least this would enable a more accurate determination of the broad AFI group.

The abridged VA instrument achieved high diagnostic accuracy for adult deaths, with high sensitivity and specificity for communicable diseases and NCDs surpassing those reported by Misganaw *et al* in a hospital-based validation study conducted in urban Ethiopia,^38^ paralleling the performance for CVD seen in previous validation studies^27 39 40^ and realising >85% overall CSMP accuracy compared with the hospital diagnoses.

The overall accuracy of the abridged VA instrument was 80%, which is higher than the 69–75% range reported by Benara *et al* using the 2016 WHO VA tool.^31^ The percentage of unspecified cases (3.3–6.7%) was similar to the 5–10% typically reported in large-scale VA studies.^20^ This instrument holds potential for enhancing COD reporting in resource-limited settings, providing a valuable tool for public health surveillance and intervention planning. While we designed the abridged questionnaire specifically to support our use of the EAVA analysis method, adaptations of other commonly used computer coded VA methods such as InterVA and InSilicoVA, as well as physician-coded VA, may also perform well with this questionnaire.^28 35 40 41^ These methods are currently used by many sites in conjunction with the WHO VA questionnaire, so demonstrating their acceptable performance together with our shortened questionnaire may further promote its use in resource-constrained settings. Such assessment was beyond the scope of the current study but could be pursued by other researchers interested in the use of these other VA analysis methods.

Investigators planning on using our abridged VA instrument (or any other VA questionnaire) should be aware that disease mix can influence VA performance^42^ including sensitivity and specificity and especially PPV and NPV. This highlights the importance of carefully considering the study setting, including hospital level, when extrapolating hospital-based study findings to a community setting. Surveillance at 16 tertiary care hospitals throughout India found that infection was the third leading cause of neonatal death, after prematurity and birth asphyxia,^43^ whereas at a subdistrict hospital in Haryana during the same time frame, there were as many neonatal deaths due to septicaemia as from prematurity and birth asphyxia combined,^44^ similar to the mortality pattern found in a community study in rural Maharashtra.^45^ These findings suggest that disease mix at lower-level hospitals may more accurately reflect the community mix than that at higher levels. However, this may not always hold true. The most common medical CODs among neonates in our study were sepsis, IPRE and pneumonia. Although our study was conducted of deaths identified at tertiary level hospitals, they all predominately serve urban slum populations, which may account for our similar findings to a Bangladeshi study conducted in urban slums using the 2016 WHO tool, which reported birth asphyxia, pneumonia and various infections as the most common causes.^46^

## Limitations

VA is most commonly used to ascertain causes of death in community settings. Our objective is to develop a tool that is both reliable and feasible for use in humanitarian crises without imposing excessive time burdens. The current findings are based on an urban poor population in Kolkata, India, and require validation in more complex, crisis-affected settings, particularly among displaced and refugee populations. Key methodological limitations that should inform interpretation of these results are outlined below.

The use of hospital COD records as the reference standard may introduce bias when extrapolating to VA performance in the community, as these records can be incomplete or inaccurate due to selective access to care, variations in treatment outcomes and the socioeconomic profiles of hospital users. Also, as discussed above, hospital level may influence disease mix. Additionally, the accuracy of the hospital diagnoses can also be influenced by physicians’ training, local diagnostic practices and the availability of resources. Snow *et al* have highlighted these inherent biases associated with relying on hospital-based data to validate VA methods.^45^ Additionally, because interviews were conducted sometime after death, recall bias is possible. However, for neonatal and child deaths, respondents were mainly parents, for whom such events are typically well remembered, potentially minimising major errors. Furthermore, limitations related to the training and knowledge of VA interviewers may contribute to misclassification or under-reporting of certain causes of death. We dealt with this limitation by selecting experienced VA interviewers and providing a 3-day training covering all aspects of the VA interview process. Finally, there were no hospital deaths from several causes that the VA is designed to test for, including neonatal tetanus, meningitis and sudden unexplained infant death, child AIDS, measles, pertussis and malaria, adult injury, tetanus, pulmonary TB+AIDS, chronic obstructive pulmonary disease, malaria and maternal haemorrhage, obstructed labour and puerperal sepsis.

## Conclusion

Wherever medical certification is not available or performing poorly, the VA approach provides a workable substitute with an overall accuracy of 80% across all age groups in our study. Our abridged VA instrument, consisting of 61 or fewer questions per age group, compared with hundreds of questions in the 2022 WHO VA questionnaire, is especially suitable for resource limited humanitarian settings. Designed for use with the EAVA analysis method, neonatal and child causes of death were diagnosed with generally high accuracy. For adult causes, the VA performed particularly well for broad disease categories. The abridged questionnaire’s performance with adaptations of other analytic methods that many sites are familiar with such as InterVA, InSilicoVA and physician coding should be assessed, and the questionnaire should be tested in other settings with common causes of death that were missing from our sample. Algorithm improvements for diseases like sepsis and other overlapping infections may improve its performance for specific conditions and facilitate even better-informed mortality surveillance and public health actions in resource-limited settings and make it better suited for integration into routine public health surveillance systems.

## Supplementary material

10.1136/bmjgh-2025-022829 online supplemental appendix 1

10.1136/bmjgh-2025-022829 online supplemental figure 1

10.1136/bmjgh-2025-022829 online supplemental file 1

## Data Availability

Data are available upon reasonable request.

## References

[1] United Nations (2025). Sustainable development goals. https://sdgs.un.org/goals.

[2] Setel PW, Macfarlane SB, Szreter S (2007). A scandal of invisibility: making everyone count by counting everyone. Lancet.

[3] Mahapatra P, Shibuya K, Lopez AD (2007). Civil registration systems and vital statistics: successes and missed opportunities. Lancet.

[4] Yokobori Y, Obara H, Sugiura Y (2021). Gaps in the civil registration and vital statistics systems of low- and middle-income countries and the health sector’s role in improving the situation. *Glob Health Med*.

[5] Guha-Sapir D, Checchi F (2018). Science and politics of disaster death tolls. BMJ.

[6] Adair T, Mikkelsen L, Hooper J (2023). Assessing the policy utility of routine mortality statistics: a global classification of countries. Bull World Health Organ.

[7] Office of the Registrar General & Census Commissioner, India Vital statistics of india based on the civil registration system 2019. https://censusindia.gov.in/.

[8] Office of the Registrar General & Census Commissioner, India Annual report on medical certification of cause of death 2020. https://dc.crsorgi.gov.in/assets/download/Annual-Reports/mccd/2020.pdf.

[9] Kumar GA, Dandona L, Dandona R (2019). Completeness of death registration in the Civil Registration System, India (2005 to 2015). Indian J Med Res.

[10] Kanungo S, Tsuzuki A, Deen JL (2010). Use of verbal autopsy to determine mortality patterns in an urban slum in Kolkata, India. Bull World Health Organ.

[11] Byass P (2007). Who needs cause-of-death data?. *PLoS Med*.

[12] Gajalakshmi V, Peto R (2004). Verbal autopsy of 80,000 adult deaths in Tamilnadu, South India. BMC Public Health.

[13] Thomas L-M, D’Ambruoso L, Balabanova D (2018). Verbal autopsy in health policy and systems: a literature review. BMJ Glob Health.

[14] Chandramohan D, Fottrell E, Leitao J (2021). Estimating causes of death where there is no medical certification: evolution and state of the art of verbal autopsy. Glob Health Action.

[15] Kalter HD, Roubanatou A-M, Koffi A (2015). Direct estimates of national neonatal and child cause-specific mortality proportions in Niger by expert algorithm and physician-coded analysis of verbal autopsy interviews. J Glob Health.

[16] Herbst AJ, Mafojane T, Newell ML (2011). Verbal autopsy-based cause-specific mortality trends in rural KwaZulu-Natal, South Africa, 2000-2009. Popul Health Metr.

[17] World Health Organization Verbal autopsy standards: the 2022 who verbal autopsy instrument. https://cdn.who.int/media/docs/default-source/classification/other-classifications/autopsy/2022-va-instrument/verbal-autopsy-standards_2022-who-verbal-autopsy-instrument_v1_final.pdf.

[18] World Health Organization (2023). Manual for the training of master trainers and supervisors on the use of the 2022 WHO VA instrument. https://cdn.who.int/media/docs/default-source/classification/other-classifications/autopsy/2022-va-instrument/2022-who-va-manual-for-supervisors-designed.pdf?sfvrsn=a1e6e007_5&download=true.

[19] Yoshida T, Fan TS, McCormick TH (2023). Bayesian active questionnaire design for cause-of-death assignment using verbal autopsies. Proc Mach Learn Res.

[20] Chandramohan D, Maude GH, Rodrigues LC (1998). Verbal autopsies for adult deaths: their development and validation in a multicentre study. Trop Med Int Health.

[21] Serina P, Riley I, Stewart A (2015). Improving performance of the Tariff Method for assigning causes of death to verbal autopsies. BMC Med.

[22] Serina P, Riley I, Stewart A (2015). A shortened verbal autopsy instrument for use in routine mortality surveillance systems. BMC Med.

[23] Centers for Disease Control and Prevention (CDC) (2014). Pertussis / whooping cough (bordetella pertussis) 2014 case definition. https://ndc.services.cdc.gov/case-definitions/pertussis-2014/.

[24] Kalter HD, Perin J, Black RE (2016). Validating hierarchical verbal autopsy expert algorithms in a large data set with known causes of death. J Glob Health.

[25] Youden WJ (1950). Index for rating diagnostic tests. Cancer.

[26] Murray CJ, Lozano R, Flaxman AD (2011). Robust metrics for assessing the performance of different verbal autopsy cause assignment methods in validation studies. Popul Health Metr.

[27] StataCorp

[28] Kaluvu L, Asogwa OA, Marzà-Florensa A (2022). Multimorbidity of communicable and non-communicable diseases in low- and middle-income countries: A systematic review. J Multimorb Comorb.

[29] Ndubuisi NE (2021). Noncommunicable Diseases Prevention In Low- and Middle-Income Countries: An Overview of Health in All Policies (HiAP). *INQUIRY*.

[30] Shrivastav R, Rawal T, Kataria I (2023). Accelerating policy response to curb non-communicable diseases: an imperative to mitigate the dual public health crises of non-communicable diseases and COVID-19 in India. *Lancet Reg Health Southeast Asia*.

[31] Benara SK, Sharma S, Juneja A (2023). Evaluation of methods for assigning causes of death from verbal autopsies in India. *Front Big Data*.

[32] Soleman N (2006). Verbal autopsy. Bull World Health Organ.

[33] Aggarwal AK, Kumar P, Pandit S (2013). Accuracy of WHO verbal autopsy tool in determining major causes of neonatal deaths in India. PLoS One.

[34] Soofi SB, Ariff S, Khan U (2015). Diagnostic accuracy of WHO verbal autopsy tool for ascertaining causes of neonatal deaths in the urban setting of Pakistan: a hospital-based prospective study. BMC Pediatr.

[35] Murray CJ, Lopez AD, Black R (2011). Population Health Metrics Research Consortium gold standard verbal autopsy validation study: design, implementation, and development of analysis datasets. Popul Health Metr.

[36] Nichols EK, Byass P, Chandramohan D (2018). The WHO 2016 verbal autopsy instrument: An international standard suitable for automated analysis by InterVA, InSilicoVA, and Tariff 2.0. PLoS Med.

[37] Leitao J, Chandramohan D, Byass P (2013). Revising the WHO verbal autopsy instrument to facilitate routine cause-of-death monitoring. Glob Health Action.

[38] Misganaw A, Mariam DH, Araya T (2012). Validity of verbal autopsy method to determine causes of death among adults in the urban setting of Ethiopia. BMC Med Res Methodol.

[39] Gomes M, Begum R, Sati P (2017). Nationwide Mortality Studies To Quantify Causes Of Death: Relevant Lessons From India’s Million Death Study. Health Aff.

[40] Murray CJL, Lopez AD, Feehan DM (2007). Validation of the symptom pattern method for analyzing verbal autopsy data. *PLoS Med*.

[41] Byass P, Hussain-Alkhateeb L, D’Ambruoso L (2019). An integrated approach to processing WHO-2016 verbal autopsy data: the InterVA-5 model. BMC Med.

[42] Bang AT, Paul VK, Reddy HM (2005). Why do neonates die in rural Gadchiroli, India? (Part I): primary causes of death assigned by neonatologist based on prospectively observed records. J Perinatol.

[43] (1997). Neonatal morbidity and mortality. Report of the National Neonatal-Perinatal Database. Indian Pediatr.

[44] Kumar M, Paul VK, Kapoor SK (2002). Neonatal outcomes at a subdistrict hospital in north India. J Trop Pediatr.

[45] Snow RW, Armstrong JR, Forster D (1992). Childhood deaths in Africa: uses and limitations of verbal autopsies. Lancet.

[46] Billah MA, Islam MZ, Chowdhury R (2024). Causes of under-five mortality using verbal autopsies in urban slum areas in Bangladesh: a cross-sectional analysis of surveillance data. Journal of Global Health Reports.

