# Supplementary material for: Determining causes of death through an abridged verbal autopsy tool: a pilot test conducted among vulnerable communities in Kolkata, India
Source: BMJ Glob Health. 2026 Jul 20;11(7):e022829. doi: 10.1136/bmjgh-2025-022829 (PMC13386064; doi:10.1136/bmjgh-2025-022829)
Supplement: online supplemental appendix 1 [file bmjgh-11-7-s001.pdf]

Appendix 1: Neonatal Verbal autopsy questionnaire

Details of respondent

N1. Name of the respondent ☐ Subject ID ☐☐☐☐☐☐

N2. Relationship of respondent with deceased **(mother will be considered as the best respondent and if she is not available in the first visit, the interviewer should make a return visit before proceeding with other suitable respondents)**

☐ 1. Mother

☐ 2. Father

☐ 3. Brother/Sister

☐ 4. Grandfather/Grandmother

☐ 5. Other close relatives

N3. Did the respondent live with the deceased during the events that led to death?

☐ 1.Yes ☐ 2. No ☐ 99. don't know ☐88. Refused to answer

N4. Respondent's age in completed years ☐☐

(Use 99 for 'Don't know' and 88 for 'Refused to answer')

N5. Respondent's sex? ☐ 1.Male ☐ 2. Female ☐ 3. Ambiguous/Intersex ☐ 99. Don't know

☐ 88. Refused to answer

N6. What is the highest standard of education the respondent has completed?

☐ 0. Illiterate and literate with no formal education

☐ 1. Literate, Primary or below

☐ 2. Literate, Middle

☐ 3. Literate, Matric Class – X

☐ 4. Literate, Class XII

☐ 5. Graduate and above

☐ 99. Don't know

☐ 88. Refused to answer

Details of deceased

N7. Sex ☐ 1. Male ☐ 2. Female ☐ 3. Ambiguous/Intersex ☐ 99. Don't know

☐ 88. Refused to answer

N8. Date of birth ☐☐ / ☐☐ / ☐☐☐☐

(Use 9 in all eight boxes if 'Don't know' and use 8 if 'Refused to answer')

N9. Date of death ☐☐ / ☐☐ / ☐☐☐☐

(Use 9 in all eight boxes if 'Don't know' and use 8 if 'Refused to answer')

N10. Age of the deceased at the time of death as stated by the respondent? ☐☐ (in days)

(Use 99 if 'Don't know' and 88 if 'Refused to answer')

(Less than 24 hours = 00 days)

If the age is greater than 27 days, then use the child questionnaire.

N11. Place of death?

- ☐ 1. Home
- ☐ 2. On way to health facility
- ☐ 3. PHC/CHC/Rural Hospital
- ☐ 4. District/Sub-divisional Hospital
- ☐ 5. State tertiary care hospital
- ☐ 6. Private Hospital
- ☐ 7. Other place
- ☐ 99. Don't know
- ☐ 88. Refused to answer

N12.A. House address of the deceased

N12. B. PIN ☐☐☐☐☐☐

N12. C. How many years did the deceased's family live at this address? ☐☐

(Use 99 for 'Don't know' and 88 for 'Refused to answer')

<table>
<tr><th colspan="2">Now I have some questions about the child's illness</th></tr>
<tr><td>N13. Was the child born alive or dead?</td><td>☐ 1. Alive ☐ 2. Dead ☐ 3. don't know
☐ 4. refused to answer</td></tr>
<tr><td>N14. Did the baby ever cry?</td><td>☐ 1. Yes ☐ 2. No ☐ 3. don't know
☐ 4. refused to answer</td></tr>
<tr><td>N15. Did the baby ever move after being delivered?</td><td>☐ 1. Yes ☐ 2. No ☐ 3. don't know
☐ 4. refused to answer</td></tr>
<tr><td>N16. Did the baby ever breathe?</td><td>☐ 1. Yes ☐ 2. No ☐ 3. don't know
☐ 4. refused to answer</td></tr>
<tr><th colspan="2">Birth status determination</th></tr>
<tr><td>N16. a.
Why do this: To determine whether this was a live birth or a stillbirth.

How to do it: The information from the above four questions must be considered together to determine the child's birth status ("Live birth" or "Stillbirth"). Refer to N13–N16:

If N13 = "1" ("Alive") and <u>ANY</u> of N14-N16 = "1" ("Yes") then mark "1" ("Live birth") and continue the interview.

If N13 is = "1" ("Alive") and <u>ALL</u> of N14-N16 = "2," ("No"), then discuss this with the respondent and correct the answer either to N13 or N14-N16.

If N13 = "2" ("Dead") and <u>ANY</u> of N14-N16 = "1" ("Yes"), then discuss this with the respondent and correct the answer either to N13 or N14-N16.

If N13 = "2" ("Dead") and <u>ALL</u> of N14-N16 = "2" ("No") then mark "2" ("Stillbirth") and end the interview.</td><td>Was it a live birth or a still birth?

☐ 1. Live birth 2. Stillbirth</td></tr>
<tr><th colspan="2">If 'dead' AND 'no cry', 'no move' AND 'no breathe', then this is a stillbirth (end interview)</th></tr>
<tr><td>N17. How many months long was the pregnancy before the child was born? (ask up to 1 year)</td><td>☐☐ Months
(Use 99 for 'Don't know' and 88 for 'Refused to answer')</td></tr>
<tr><td>N18. Were there any bruises or signs of injury on the baby's body at birth?</td><td>☐ 1. Yes ☐ 2. No ☐ 3. don't know
☐ 4. refused to answer</td></tr>
<tr><td>N19. Was any part of the baby physically abnormal at the time of delivery? (for example: body part too large or too small, additional growth on body)</td><td>☐ 1. Yes ☐ 2. No ☐ 3. don't know
☐ 4. refused to answer</td></tr>
<tr><td>N20. Did the baby/ child have a swelling or defect on the back at time of birth?</td><td>☐ 1. Yes ☐ 2. No ☐ 3. don't know
☐ 4. refused to answer</td></tr>
<tr><td>N21. Did the baby/ child have a very large head at time of birth?</td><td>☐ 1. Yes ☐ 2. No ☐ 3. don't know
☐ 4. refused to answer</td></tr>
<tr><th colspan="2">If yes, skip to question N23</th></tr>
</table>

N22. Did the baby/ child have a very small head at time of birth?	☐ 1. Yes ☐ 2. No ☐ 3. don’t know<br>☐ 4. refused to answer
N23. Did the baby breathe immediately after birth, even a little?	☐ 1. Yes ☐ 2. No ☐ 3. don’t know<br>☐ 4. refused to answer
N24. Was anything done to try to help the baby breathe at birth?	☐ 1. Yes ☐ 2. No ☐ 3. don’t know<br>☐ 4. refused to answer
N25. Did the baby cry immediately after birth, even if only a little bit?	☐ 1. Yes ☐ 2. No ☐ 3. don’t know<br>☐ 4. refused to answer
**If yes, skip to question N27**	
N26. How many minutes after birth did the baby first cry?	☐☐☐ (use 998 if never cried)<br>(Use 999 if ‘Don’t know’ and 888 if ‘Refused to answer’)
N27. Did the baby stop being able to cry?	☐ 1. Yes ☐ 2. No ☐ 3. don’t know<br>☐ 4. refused to answer
**If ‘No’, ‘Don’t know’, or ‘Refused to answer’ skip to question N29**	
N28. How many days after birth did the baby stop being able to cry?	☐☐ Days (Less than 24 hours = 00 days)<br>(Use 99 if ‘Don’t know’ and 88 if ‘Refused to answer)
N29. Was the baby able to suckle or bottle-feed in a normal way during the first day of life?	☐ 1. Yes ☐ 2. No ☐ 3. don’t know<br>☐ 4. refused to answer
**If yes, skip to question N31**	
N30. Did the baby ever suckle in a normal way?	☐ 1. Yes ☐ 2. No ☐ 3. don’t know<br>☐ 4. refused to answer
**If ‘**No’, ‘Don’t know’, or ‘Refused to answer**, skip to question N33**	
N31. Did the baby stop being able to suckle in a normal way?	☐ 1. Yes ☐ 2. No ☐ 3. don’t know<br>☐ 4. refused to answer
**If ‘**No’, ‘Doesn’t know’, or ‘Refused to answer**, skip to question N33**	
N32. How many days after birth did the baby stop suckling?	☐☐ Days (Less than 24 hours = 00 days)<br>(Use 99 if ‘Don’t know’ and 88 if ‘Refused to answer’)
N33. During the illness that led to death, did the baby have spasms or convulsions?	☐ 1. Yes ☐ 2. No ☐ 3. don’t know<br>☐ 4. refused to answer
**If ‘**No’, ‘Don’t know’, or ‘Refused to answer’**, skip to question N36**	
N34. Did the baby have convulsions starting in the first 24 hours of life?	☐ 1. Yes ☐ 2. No ☐ 3. don’t know<br>☐ 4. refused to answer
**If yes, skip to question N36**	
N35. Did the baby have convulsions starting more than 24 hours after birth?	☐ 1. Yes ☐ 2. No ☐ 3. don’t know<br>☐ 4. refused to answer
N36. During the illness that led to death, did the baby become unresponsive or unconscious?	☐ 1. Yes ☐ 2. No ☐ 3. don’t know<br>☐ 4. refused to answer
N37. During the illness that led to death, did the baby become lethargic, after a period of normal activity?	☐ 1. Yes ☐ 2. No ☐ 3. don’t know<br>☐ 4. refused to answer
N38. During the illness that led to death, did the baby have pus drainage from the umbilical cord stump?	☐ 1. Yes ☐ 2. No ☐ 3. don’t know<br>☐ 4. refused to answer
N39. During the illness that led to death, did the baby have redness of the umbilical cord stump?	☐ 1. Yes ☐ 2. No ☐ 3. don’t know<br>☐ 4. refused to answer

**If 'No', 'Don't know', or 'Refused to answer', skip to question N41**	
N40. Did the redness of the umbilical cord stump extend onto the abdominal skin?	☐ 1. Yes ☐ 2. No ☐ 3. don't know<br>☐ 4. refused to answer
N41. During the illness that led to death, did the baby have skin ulcer(s) or pit(s)?	☐ 1. Yes ☐ 2. No ☐ 3. don't know<br>☐ 4. refused to answer
N42. During the illness that led to death, did the baby have fever?	☐ 1. Yes ☐ 2. No ☐ 3. don't know<br>☐ 4. refused to answer
N43. During the illness that led to death, did the baby become cold to touch?	☐ 1. Yes ☐ 2. No ☐ 3. don't know<br>☐ 4. refused to answer
N44. During the illness that led to death, did the baby have difficulty breathing?	☐ 1. Yes ☐ 2. No ☐ 3. don't know<br>☐ 4. refused to answer
**If 'No', 'Don't know', or 'Refused to answer', skip to question N46**	
N45. A. For how many days did the difficult breathing last?	☐☐ Days (Less than 24 hours = 00 days)<br>(Use 99 if 'Don't know' and 88 if 'Refused to answer')
N45. B. Did the difficult breathing last until the baby died?	☐ 1. Yes ☐ 2. No ☐ 3. don't know<br>☐ 4. refused to answer
N46. During the illness that led to death, did the baby have fast breathing?	☐ 1. Yes ☐ 2. No ☐ 3. don't know<br>☐ 4. refused to answer
**If 'No', 'Don't know', or 'Refused to answer, skip to question N49**	
N47. At what age did the fast-breathing start?	☐☐ days (Less than 24 hours = 00 days)<br>(Use 99 if 'Don't know' and 88 if 'Refused to answer')
N48. For how many days did the fast breathing last?	☐☐ days (Less than 24 hours = 00 days)<br>(Use 99 if 'Don't know' and 88 if 'Refused to answer')
N49. During the illness that led to death, did you see the lower chest wall/ribs being pulled in as the baby breathed?	☐ 1. Yes ☐ 2. No ☐ 3. don't know<br>☐ 4. refused to answer
N50. During the illness that led to death, did her/his breathing sound like this?<br>(Demonstrate the grunting sound to the respondent)	☐ 1. Yes ☐ 2. No<br>☐ 3. Don't know ☐ 4.refused to answer
N51. Did the baby have a bulging or raised fontanelle during the illness that led to death? (Show photo)	☐ 1. Yes ☐ 2. No ☐ 3. don't know<br>☐ 4. refused to answer
N52. During the illness that led to death, did s/he have yellow skin, palms (hand) or soles (foot)?	☐ 1. Yes ☐ 2. No ☐ 3. don't know<br>☐ 4. refused to answer
N53. During the illness that led to death, did the baby have yellow discoloration of the eyes?	☐ 1. Yes ☐ 2. No ☐ 3. don't know<br>☐ 4. refused to answer
N54. During the illness that led to death, did the baby have an area(s) of skin with redness and swelling?	☐ 1. Yes ☐ 2. No ☐ 3. don't know<br>☐ 4. refused to answer
N55. During the illness that led to death, did s/he have areas of the skin that turned black?	☐ 1. Yes ☐ 2. No ☐ 3. don't know<br>☐ 4. refused to answer
N56. During the illness that led to death, did the baby bleed from anywhere?	☐ 1. Yes ☐ 2. No ☐ 3. don't know<br>☐ 4. refused to answer

N57. During the illness that led to death, did s/he have more frequent loose or liquid stools than usual?	☐ 1. Yes ☐ 2. No ☐ 3. don't know<br>☐ 4. refused to answer
**If '**No', 'Don't know', or 'Refused to answer'**, skip to question N59**	
N58. How many stools did the baby have on the day that loose liquid stools were most frequent?	☐☐ number of times<br>(Use 99 if 'Don't know' and 88 if 'Refused to answer')
N59. During the illness that led to death, did the baby vomit?	☐ 1. Yes ☐ 2. No ☐ 3. don't know<br>☐ 4. refused to answer
**If '**No', 'Don't know', or 'Refused to answer'**, skip to question N61**	
N60. Did s/he vomit every time s/he drank?	☐ 1. Yes ☐ 2. No ☐ 3. don't know<br>☐ 4. refused to answer
N61. Did the baby appear to be healthy and then just die suddenly? (Suddenly means died unexpectedly within 24 hours of being in regular health)	☐ 1. Yes ☐ 2. No ☐ 3. don't know<br>☐ 4. refused to answer

N62. What did the respondent think that this person died of? (Allow the respondent to tell the illness in his or her own words)

END OF INTERVIEW.

THANK RESPONDENT FOR HER/HIS PARTICIPATION.

Interviewer: Use the below space to write down your comments and observations about the interview, including:

1. Did you have any trouble locating the deceased's household or identifying the best respondent?

2. Did the respondent understand all/most of the questions?

3. Was the respondent interested in the interview and try to answer the questions accurately?

4. Do you have any other comments?

Appendix 2: Child Verbal autopsy questionnaire

Details of respondent

C1. Name of the respondent ☐ Subject ID ☐☐☐☐☐☐

C2. Relationship of respondent with deceased (**(mother will be considered as the best respondent and if she is not available in the first visit, the interviewer should make a return visit before proceeding with other suitable respondents)**

☐ 1. Brother/Sister

☐ 2. Mother

☐ 3. Father

☐ 4. Grandfather/Grandmother

☐ 5. Other close relatives

C3. Did the respondent live with the deceased during the events that led to death?

☐ 1.Yes ☐ 2. No ☐ 99. Don't know ☐ 88. Refused to answer

C4. Respondent's age in completed years ☐☐

(Use 99 for 'Don't know' and 88 for 'Refused to answer')

C5. Respondent's sex? ☐ 1.Male ☐ 2. Female ☐ 3. Ambiguous/Intersex ☐ 99. Don't know

☐ 88. 'Refused to answer'

C6. What is the highest standard of education the respondent has completed?

☐ 0. Illiterate and literate with no formal education

☐ 1. Literate, Primary or below

☐ 2. Literate, Middle

☐ 3. Literate, Matric Class – X

☐ 4. Literate, Class XII

☐ 5 Graduate and above

☐ 99. Don't know

☐ 88. Refused to answer

Details of deceased

C7. Sex ☐ 1. Male ☐ 2. Female ☐ 3. Ambiguous/Intersex ☐ 99. Don't know

☐ 88. Refused to answer

C8. Date of birth ☐☐/☐☐/☐☐☐☐

(Use 9 in all eight boxes if 'Don't know' and 8 if 'Refused to answer')

C9. Date of death ☐☐/☐☐/☐☐☐☐

(Use 9 in all eight boxes if 'Don't know' and 8 if 'Refused to answer')

C10. A. Age of Deceased as stated by the respondent ☐ 1. Months ☐ 2. Years

☐ 3. Don't know ☐ 4. Refused to answer

If don't know or Refused to answer, skip to C11

C10. B. How many months/ years? ☐☐ (Use 99 for 'Don't know' and 88 for 'Refused to answer')

(Record months if 1-23 months; Record years if 2-11 years; If 12 or more years, use Adult questionnaire.

C11. What is the highest standard of education the deceased has completed?

☐ 0. Illiterate and literate with no formal education

☐ 1. Literate, Primary or below ☐ 88. Refused to answer

☐ 2. Literate, Middle

☐ 3. Literate, Matric Class – X

☐ 4. Literate, Class XII

☐ 99. Don't know

C12. Place of death?

- ☐ 1. Home
- ☐ 2. On way to health facility
- ☐ 3. PHC/CHC/Rural Hospital
- ☐ 4. District/Sub-divisional Hospital
- ☐ 5. State tertiary care hospital
- ☐ 6. Private Hospital
- ☐ 7. Other place
- ☐ 99. Don't know
- ☐ 88. Refused to answer

C13. A. House address of the deceased

C13. B. PIN ☐☐☐☐☐☐

C13. C. How many years did the deceased's family live at this address? ☐☐

(Use 99 for 'Don't know' and 88 for 'Refused to answer')

Now I have some questions about the child's illness.	
C14. A. For how long was (s)he ill before death?	☐ 1. Days ☐ 2. Months ☐ 3. Years<br>☐ 4. Don't know ☐ 5. Refused to answer
If don't know or Refused to answer, skip to C15	
C14. B. For how many days/ months/ years was (s)he ill?	☐☐<br>Record days if less than 28 days; less than 24 hours = 00 days.<br>(Use 99 for 'Don't know' and 88 for 'Refused to answer')
**Questions C15 to C29 are for deaths of children 1-11 months of age. If 1 year or older, skip to C30**	
C15. How many months long was the pregnancy before birth?	☐☐ Months<br>(Use 99 for 'Don't know' and 88 to 'Refused to answer')
C16. Was any part of the baby physically abnormal at the time of delivery? (for example: body part too large or too small, additional growth on body)	☐ 1. Yes ☐ 2. No ☐ 3. don't know<br>☐ 4. refused to answer

C17. Did the baby/ child have a swelling or defect on the back at time of birth?	☐ 1. Yes ☐ 2. No ☐ 3. don't know ☐ 4. refused to answer
C18. Did the baby/ child have a very large head at time of birth?	☐ 1. Yes ☐ 2. No ☐ 3. don't know ☐ 4. refused to answer
**If Yes, skip to question C20**	
C19. Did the baby/ child have a very small head at time of birth?	☐ 1. Yes ☐ 2. No ☐ 3. don't know ☐ 4. refused to answer
C20. Did the baby breathe immediately after birth, even a little?	☐ 1. Yes ☐ 2. No ☐ 3. don't know ☐ 4. refused to answer
C21. Did the baby cry immediately after birth, even if only a little bit?	☐ 1. Yes ☐ 2. No ☐ 3. don't know ☐ 4. refused to answer
**If Yes, skip to question C23**	
C22. How many minutes after birth did the baby first cry?	☐☐☐ Minutes (use 998 if never cried) (Use 999 if 'Don't know' and 888 if 'Refused to answer')
C23. Was the baby able to suckle or bottle-feed within the first 24 hours after birth?	☐ 1. Yes ☐ 2. No ☐ 3. don't know ☐ 4. refused to answer
C24. During the illness that led to death, did the baby become cold to touch?	☐ 1. Yes ☐ 2. No ☐ 3. don't know ☐ 4. refused to answer
C25. During the illness that led to death, did the baby have convulsions?	☐ 1. Yes ☐ 2. No ☐ 3. don't know ☐ 4. refused to answer
**If 'No', 'Don't know' or 'Refused to answer' skip to question C28**	
C26. Did the baby have convulsions starting in the first 24 hours of life?	☐ 1. Yes ☐ 2. No ☐ 3. don't know ☐ 4. refused to answer
**If Yes, skip to question C28**	
C27. Did the baby have convulsions starting more than 24 hours after birth?	☐ 1. Yes ☐ 2. No ☐ 3. don't know ☐ 4. refused to answer
C28. During the illness that led to death, did the baby become lethargic, after a period of normal activity?	☐ 1. Yes ☐ 2. No ☐ 3. don't know ☐ 4. refused to answer
C29. Did the baby have a bulging or raised fontanelle during the illness that led to death?	☐ 1. Yes ☐ 2. No ☐ 3. don't know ☐ 4. refused to answer
**All the remaining questions are for deaths of children of all ages**	
C30. During the illness that led to death, did child have a fever?	☐ 1. Yes ☐ 2. No ☐ 3. don't know ☐ 4. refused to answer
If 'No', 'Don't know', or 'Refused to answer', skip to question C31	

a) i. How long did the fever last?	☐ 1. Days ☐ 2. Months<br>☐ 3. don't know ☐ 4. refused to answer
If 'Don't know' or 'Refused to answer', skip to question C30. b)	
a) ii. How many days/ months did the fever last?	☐☐<br>(Use 99 for 'Don't know' and 88 for 'Refused to answer')
b) Did the fever continue until death?	☐ 1. Yes ☐ 2. No ☐ 3. don't know<br>☐ 4. refused to answer
c) How severe was the fever?	☐ 1. mild ☐ 2. moderate ☐ 3. severe<br>☐ 4. don't know ☐ 5. refused to answer
d) What was the pattern of the fever?	☐ 1. continuous ☐ 2. on and off<br>☐ 3. only at night ☐ 4. don't know<br>☐ 5. refused to answer
C31. During the illness that led to death, did he/she have more frequent loose or liquid stools than usual?	☐ 1. Yes ☐ 2. No ☐ 3. don't know<br>☐ 4. refused to answer
If 'No', 'Don't know', or 'Refused to answer', skip to question C32	
a) i. How long did the frequent loose or liquid stools last?	☐ 1. Days ☐ 2. Weeks<br>☐ 3. don't know ☐ 4. refused to answer
If 'Don't know' or 'Refused to answer', skip to question C31. b)	
a) ii. How many days/weeks did the frequent loose or liquid stools last?	☐☐<br>(Use 99 for Don't know and 88 for Refused to answer)
b) How many stools did he/she have on the day that loose liquid stools were most frequent?	☐☐ number of times<br>(Use 99 for Don't know and 88 for Refused to answer)
c) At any time during the fatal illness was there visible blood in the loose or liquid stools?	☐ 1. Yes ☐ 2. No ☐ 3. don't know<br>☐ 4. refused to answer
C32. Did s/he have a more than usually protruding abdomen?	☐ 1. Yes ☐ 2. No ☐ 3. don't know<br>☐ 4. refused to answer
C33. During the illness that led to death, did the child have a cough?	☐ 1. Yes ☐ 2. No ☐ 3. don't know<br>☐ 4. refused to answer
If 'No', 'Don't know', or 'Refused to answer', skip to question C34	
a) i. For how long did s/he have a cough?	☐ 1. Days ☐ 2. Months ☐ 3. don't know<br>☐ 4. refused to answer

**If 'Don't know' or 'Refused to answer', skip to question C33. b)**	
a) ii. For how many days/months did s/he have a cough?	☐☐ (Use 99 for Don't know and 88 for Refused to answer)
b) Was the cough very severe?	☐ 1. Yes ☐ 2. No ☐ 3. don't know ☐ 4. refused to answer
c) Did the child make a whooping sound when coughing?	☐ 1. Yes ☐ 2. No ☐ 3. don't know ☐ 4. refused to answer
d) Did the child vomit after s/he coughed?	☐ 1. Yes ☐ 2. No ☐ 3. don't know ☐ 4. refused to answer
C34. During the illness that led to death, did child have difficulty breathing?	☐ 1. Yes ☐ 2. No ☐ 3. don't know ☐ 4. refused to answer
**If 'No', 'don't know' or 'Refused to answer' skip to question C36**	
C35. a) For how long did the difficult breathing last?	☐ 1. Days ☐ 2. Months ☐ 3. Years ☐ 4. don't know ☐ 5. refused to answer
**If 'Don't know' or 'Refused to answer', skip to question C36.**	
C35. b) For how many days/ months/ years did the difficult breathing last?	☐☐ (Use 99 for Don't know and 88 for Refused to answer)
C36. During the illness that led to death, did child have fast breathing?	☐ 1. Yes ☐ 2. No ☐ 3. don't know ☐ 4. refused to answer
**If 'No', 'don't know' or 'Refused to answer' skip to question C39**	
C37. a) At what age did the fast-breathing start?	☐ 1. Days ☐ 2.Months ☐ 3. Years ☐ 4. don't know ☐ 5. refused to answer
**If 'Don't know' or 'Refused to answer', skip to question C38. a)**	
C37. b) At what days/ months/ years did the fast-breathing start?	☐☐ (Use 99 for Don't know and 88 for Refused to answer)
C38. a) How long did the fast breathing last?	☐ 1. Days ☐ 2. Months ☐ 3. don't know ☐ 4. refused to answer
**If 'Don't know' or 'Refused to answer', skip to question C39**	
C38. b) For how many days/ months did the fast breathing last?	☐☐ (Use 99 for Don't know and 88 for Refused to answer)

C39. Ask only for children <5 years old: During the illness that led to death, did you see the lower chest wall/ribs being pulled in as the child breathed?	☐ 1. Yes ☐ 2. No ☐ 3. don't know ☐ 4. Refused to answer
C40. During the illness that led to death, did her/his breathing sound like any of the following?	
**Demonstrate each sound:**	
a. Stridor	☐ 1. Yes ☐ 2. No ☐ 3. don't know ☐ 4. refused to answer
b. Grunting	☐ 1. Yes ☐ 2. No ☐ 3. don't know ☐ 4. refused to answer
c. Wheezing	☐ 1. Yes ☐ 2. No ☐ 3. don't know ☐ 4. refused to answer
C41. Did child have a stiff or painful neck during the illness that led to death?	☐ 1. Yes ☐ 2. No ☐ 3. don't know ☐ 4. refused to answer
C42. Was child unconscious during the illness that led to death?	☐ 1. Yes ☐ 2. No ☐ 3. don't know ☐ 4. refused to answer
**If 'No', 'Don't know', or 'Refused to answer', skip to question C43**	
a) i. How long before death did unconsciousness start?	☐ 1. Hours ☐ 2. Days ☐ 3. don't know ☐ 4. refused to answer
**If 'Don't know' or 'Refused to answer', skip to question C42. b)**	
a) ii. How many hours/ days before death did unconsciousness start?	☐☐ (Use 99 for Don't know and 88 for Refused to answer)
b) Did the unconsciousness continue until death?	☐ 1. Yes ☐ 2. No ☐ 3. don't know ☐ 4. refused to answer
C43. During the illness that led to death, did child have any generalized convulsions or fits?	☐ 1. Yes ☐ 2. No ☐ 3. don't know ☐ 4. refused to answer
C44. During the month before s/he died, did child have a skin rash?	☐ 1. Yes ☐ 2. No ☐ 3. don't know ☐ 4. refused to answer
**If 'No', 'Don't know', or 'Refused to answer', skip to question C45**	
a) Where was the rash?	☐ 1.Face ☐ 2. Trunk/abdomen ☐ 3.Extremities ☐ 4. everywhere ☐ 5.don't know ☐ 6.refused to answer
b) Where did the rash start?	☐ 1.Face ☐ 2. Trunk/abdomen ☐ 3. Upper Extremities ☐ 4. Lower Extremities ☐ 5. don't know

	☐ 6.refused to answer
c) i. How long did the rash last?	☐ 1. Days ☐ 2. Months ☐ 3. don't know ☐ 4. refused to answer
**If 'Don't know' or 'Refused to answer', skip to question C45**	
c) ii. How many days/ months did the rash last?	☐☐ (Use 99 for Don't know and 88 for Refused to answer)
C45. Did child have noticeable weight loss? (became very thin)	☐ 1. Yes ☐ 2. No ☐ 3. don't know ☐ 4. refused to answer
C46. Was s/he severely thin or wasted?	☐ 1. Yes ☐ 2. No ☐ 3. don't know ☐ 4. refused to answer
C47. During the illness that led to death, did child have swollen legs or feet?	☐ 1. Yes ☐ 2. No ☐ 3. don't know ☐ 4. refused to answer
**If 'No',** 'Don't know', or 'Refused to answer' **skip to question C48**	
C47. a) How long did the swelling last?	☐ 1. Days ☐ 2. Months ☐ 3. don't know ☐ 4. refused to answer
**If 'Don't know' or 'Refused to answer', skip to question C48**	
C47. b) How many days/ months did the swelling last?	☐☐ (Use 99 for Don't know and 88 for Refused to answer)
C48. Did s/he look pale (thinning/lack of blood) or have pale palms, eyes or nail bed?	☐ 1. Yes ☐ 2. No ☐ 3. don't know ☐ 4. refused to answer
C49. Did s/he have any lumps on the armpit?	☐ 1. Yes ☐ 2. No ☐ 3. don't know ☐ 4. refused to answer
C50. During the illness that led to death, did child have a whitish rash inside the mouth or on the tongue?	☐ 1. Yes ☐ 2. No ☐ 3. don't know ☐ 4. refused to answer
C51. During the illness that led to death, did child bleed from the nose, mouth or anus?	☐ 1. Yes ☐ 2. No ☐ 3. don't know ☐ 4. refused to answer
C52. During the illness that led to death, did s/he have areas of the skin that turned black?	☐ 1. Yes ☐ 2. No ☐ 3. don't know ☐ 4. refused to answer
C53. Did child suffer from any injury or accident that led to her/his death?	☐ 1. Yes ☐ 2. No ☐ 3. don't know ☐ 4. refused to answer
**If 'No', 'don't know' or 'Refused to answer', skip to question C56**	
C54. What was the injury or accident? Was it:	

Skip to question C55. a), if any of the below questions (C54.a - C54.j) returns a 'YES' response	
a. A road traffic crash/injury?	☐ 1. Yes ☐ 2. No ☐ 3. don't know ☐ 4. refused to answer
b. A fall?	☐ 1. Yes ☐ 2. No ☐ 3. don't know ☐ 4. refused to answer
c. drowning?	☐ 1. Yes ☐ 2. No ☐ 3. don't know ☐ 4. refused to answer
d. poisoning?	☐ 1. Yes ☐ 2. No ☐ 3. don't know ☐ 4. refused to answer
e. A bite by a dog or monkey?	☐ 1. Yes ☐ 2. No ☐ 3. don't know ☐ 4. refused to answer
f. A bite or sting by a venomous animal?	☐ 1. Yes ☐ 2. No ☐ 3. don't know ☐ 4. refused to answer
g. A burn?	☐ 1. Yes ☐ 2. No ☐ 3. don't know ☐ 4. refused to answer
h. any other injury?	☐ 1. Yes ☐ 2. No ☐ 3. don't know ☐ 4. refused to answer
i. intentionally inflicted by someone else (homicide, abuse)?	☐ 1. Yes ☐ 2. No ☐ 3. don't know ☐ 4. refused to answer
j. self-inflicted?	☐ 1. Yes ☐ 2. No ☐ 3. don't know ☐ 4. refused to answer
C55. a) How long after the injury or accident did s/he die?	☐ 1. Days ☐ 2. Months ☐ 3. don't know ☐ 4. refused to answer
If 'Don't know' or 'Refused to answer', skip to question C56	
C55. b) How many days/ months after the injury or accident did s/he die?	☐☐ (Use 99 for Don't know and 88 for Refused to answer)
C56. Was there any diagnosis by a health professional that the child had AIDS?	☐ 1. Yes ☐ 2. No ☐ 3. don't know ☐ 4. refused to answer
C57. Did (you / the child's biological mother) ever have a positive HIV test?	☐ 1. Yes ☐ 2. No ☐ 3. don't know ☐ 4. refused to answer
C58. Was there any diagnosis by a health professional that (you / the child's biological mother) had AIDS?	☐ 1. Yes ☐ 2. No ☐ 3. don't know ☐ 4. refused to answer
C59. Before (her / his) fatal illness began, did child sleep under an insecticide treated bed net?	☐ 1. Yes ☐ 2. No ☐ 3. don't know ☐ 4. refused to answer

C60. Of all the symptoms that the child had during the illness, which was the first one to appear?	

C61. What did the respondent think that this person died of? (Allow the respondent to tell the illness in his or her own words

END OF INTERVIEW.

THANK RESPONDENT FOR HER/HIS PARTICIPATION.

Interviewer: Use the below space to write down your comments and observations about the interview, including:

1. Did you have any trouble locating the deceased’s household or identifying the best respondent?

2. Did the respondent understand all/most of the questions?

3. Was the respondent interested in the interview and try to answer the questions accurately?

4. Do you have any other comments?

Appendix 3: Adult verbal autopsy questionnaire

Details of respondent

A1. Name of the respondent ☐ Subject ID ☐☐☐☐☐☐

A2. Relationship of respondent with deceased

- ☐ 1. Wife/Husband
- ☐ 2. Brother/Sister
- ☐ 3. Son/Daughter
- ☐ 4. Mother/Father
- ☐ 5. Grandchild
- ☐ 6. Brother-in-law/Sister-in-Law
- ☐ 7. Parent-in-law
- ☐ 8. Grandfather/Grandmother
- ☐ 9. Other close relatives

A3. Did the respondent live with the deceased during the events that led to death?

☐ 1.Yes ☐ 2. No ☐ 99. Don't know ☐ 88. Refused to answer

A4. Respondent's age in completed years ☐☐

(Use 99 for 'Don't know' and 88 for 'Refused to answer')

A5. Respondent's sex? ☐ 1.Male ☐ 2. Female ☐ 3. Ambiguous/Intersex ☐ 99. Don't know

☐ 88. Refused to answer

A6. What is the highest standard of education the respondent has completed?

- ☐ 0. Illiterate and literate with no formal education
- ☐ 1. Literate, Primary or below
- ☐ 2. Literate, Middle
- ☐ 3. Literate, Matric Class – X
- ☐ 4. Literate, Class XII
- ☐ 5. Graduate and above
- ☐ 99. Don't know
- ☐ 88. Refused to answer

Details of deceased

A7. Sex ☐ 1. Male ☐ 2. Female ☐ 3. Ambiguous/Intersex ☐ 99. Don't know

☐ 88. Refused to answer

A8. Date of birth ☐☐ / ☐☐ / ☐☐☐☐

(Use 9 in all eight boxes if 'Don't know' and 8 if 'Refused to answer')

A9. Date of death ☐☐ / ☐☐ / ☐☐☐☐

(Use 9 in all eight boxes if 'Don't know' and 8 if 'Refused to answer')

A10. Age of Deceased as stated by the respondent ☐☐ (in years)

(Use 99 for 'Don't know' and 88 for 'Refused to answer')

A11. What is the highest standard of education the deceased has completed?

- ☐ 0. Illiterate and literate with no formal education
- ☐ 1. Literate, Primary or below
- ☐ 2. Literate, Middle
- ☐ 3. Literate, Matric Class – X
- ☐ 4. Literate, Class XII
- ☐ 5. Graduate and above
- ☐ 99. Don't know
- ☐ 88. Refused to answer

A12. What was the occupation of the deceased?

- ☐ 1. Non-worker
- ☐ 2. Salaried
- ☐ 3. Wage earner
- ☐ 4. Profession/Business
- ☐ 5. Cultivator/farmer
- ☐ 6. Agricultural wage labour
- ☐ 7. Nonagricultural wage labour
- ☐ 8. Student
- ☐ 9. Other
- ☐ 99. Don't know
- ☐ 88. Refused to answer

A13. Place of death?

- ☐ 1. Home
- ☐ 2. On way to health facility
- ☐ 3. PHC/CHC/Rural Hospital
- ☐ 4. District/Sub-divisional Hospital
- ☐ 5. State tertiary care hospital
- ☐ 6. Private Hospital
- ☐ 7. Other place
- ☐ 99. Don't know
- ☐ 88. Refused to answer

A14. A. House address of the deceased

A14. B. PIN ☐☐☐☐☐☐

A14. C. How many years did the deceased live at this address? ☐☐

(Use 99 if 'Don't know' and 88 if 'Refused to answer')

Now I have some questions about the illness.	
A15. A) For how long was (s)he ill before death?	☐ 1. Days ☐ 2. Months ☐ 3. Years<br>☐ 4. don't know ☐ 5. refused to answer

If ‘Don’t know’ or ‘Refused to answer’, skip to question A16	
A15. B) For how many days/ months/ years was (s)he ill before death?	☐☐ (Use 99 if ‘Don’t know’ and 88 if ‘Refused to answer’)
A16. Did s/he die suddenly? (Within 24 hours of being in regular health)	☐ 1. Yes ☐ 2. No ☐ 3. don’t know ☐ 4. refused to answer
A17. During the illness that led to death, did he/she have a fever?	☐ 1. Yes ☐ 2. No ☐ 3. don’t know ☐ 4. refused to answer
**If ‘NO’, ‘Don’t know’, or ‘Refused to answer’ skip to question A18**	
a) i. How long did the fever last?	☐ 1. Days ☐ 2. Months ☐ 3. don’t know ☐ 4. refused to answer
**If ‘Don’t know’ or ‘Refused to answer’, skip to question A17. b)**	
a) ii. How many days/ months did the fever last?	☐☐ (Use 99 if ‘Don’t know’ and 88 if ‘Refused to answer)
b) How severe was the fever?	☐ 1. mild ☐ 2. moderate ☐ 3. Severe ☐ 4. doesn't know ☐ 5. refused to answer
c) What was the pattern of the fever?	☐ 1. continuous ☐ 2. on and off ☐ 3. only at night ☐ 4. don’t know ☐ 5. refused to answer
A18. During the illness that led to death, did he/she have more frequent loose or liquid stools than usual?	☐ 1. Yes ☐ 2. No ☐ 3. don’t know ☐ 4. refused to answer
**If ‘NO’,** Don’t know’, or ‘Refused to answer’ **skip to question A19**	
a) i. How long did the frequent loose or liquid stools last?	☐ 1. Days ☐ 2. Weeks ☐ 3. Don’t know ☐ 4. Refused to answer
**If ‘Don’t know’ or ‘Refused to answer’, skip to question A18. b)**	
a) ii. How many days/ weeks did the frequent loose or liquid stools last?	☐☐ (Use 99 if ‘Don’t know’ and 88 if ‘Refused to answer’)
b) How many stools did he/she have on the day that loose liquid stools were most frequent?	☐☐ number of times (Use 99 for Don’t know and 88 for Refused to answer)
A19. At any time during the fatal illness, was there blood in the stools?	☐ 1. Yes ☐ 2. No ☐ 3. don’t know ☐ 4. refused to answer
A20. During the illness that led to death, did the deceased vomit?	☐ 1. Yes ☐ 2. No ☐ 3. don’t know ☐ 4. refused to answer
**If ‘NO’, ‘Don’t know’, or ‘Refused to answer’ skip to question A22**	

A21. Was the vomit bloody or black, or did it smell like feces?	☐ 1. Yes ☐ 2. No ☐ 3. don't know ☐ 4. refused to answer
A22. Did s/he has belly (abdominal) pain?	☐ 1. Yes ☐ 2. No ☐ 3. don't know ☐ 4. refused to answer
**If 'NO', 'Don't know', or 'Refused to answer' skip to question A23**	
a) Was the belly (abdominal) pain severe?	☐ 1. Yes ☐ 2. No ☐ 3. don't know ☐ 4. refused to answer
b) Did s/he faint when s/he had the pain?	☐ 1. Yes ☐ 2. No ☐ 3. don't know ☐ 4. refused to answer
A23. Did s/he have a more than usually protruding belly (abdomen)?	☐ 1. Yes ☐ 2. No ☐ 3. don't know ☐ 4. refused to answer
**If 'NO', 'Don't know', or 'Refused to answer' skip to question A24**	
a) i. For how long before death did s/he have a more than usually protruding belly (abdomen)?	☐ 1. Days ☐ 2. Months ☐ 3. Years ☐ 4. Don't know ☐ 5. Refused to answer
**If 'Don't know' or 'Refused to answer', skip to question A24**	
a) ii. For how many days/ months/years before death did s/he have more than usually protruding belly (abdomen)?	☐☐ (Use 99 if 'Don't know' and 88 if 'Refused to answer')
A24. During the illness that led to death, did the deceased have a cough?	☐ 1. Yes ☐ 2. No ☐ 3. don't know ☐ 4. refused to answer
**If 'NO', 'Don't know', or 'Refused to answer' skip to question A25**	
a) i. For how long did s/he have a cough?	☐ 1. Days ☐ 2. Months ☐ 3. Years ☐ 4. Don't know ☐ 5. Refused to answer
**If 'Don't know' or 'Refused to answer', skip to question A24. b)**	
a) ii. For how many days/ months/years did s/he have a cough?	☐☐ (Use 99 if 'Don't know' and 88 if 'Refused to answer')
b) Was the cough productive, with sputum?	☐ 1. Yes ☐ 2. No ☐ 3. don't know ☐ 4. refused to answer
**If 'NO', 'Don't know', or 'Refused to answer', skip to question A24.d)**	
c) i. For how long was the cough productive, with sputum?	☐ 1. Days ☐ 2. Months ☐ 3. Years ☐ 4. Don't know ☐ 5. refused to answer
**If 'Don't know' or 'Refused to answer', skip to question A24. d)**	
c) ii. For how many days/ months/ years was the cough productive, with sputum?	☐☐ (Use 99 if 'Don't know' and 88 if 'Refused to answer')

d) Did s/he cough up blood?	☐ 1. Yes ☐ 2. No ☐ 3. don't know<br>☐ 4. refused to answer
A25. During the illness that led to death, did he/she have difficulty breathing or breathlessness?	☐ 1. Yes ☐ 2. No ☐ 3. don't know<br>☐ 4. refused to answer
**If 'NO', 'Don't know', or 'Refused to answer' skip to question A26**	
a) i. For how long did the difficult breathing or breathlessness last?	☐ 1. Days ☐ 2. Months ☐ 3. Years<br>☐ 4. Don't know ☐ 5. Refused to answer
**If 'Don't know' or 'Refused to answer', skip to question A25. b)**	
a) ii. For how many days/ months/ years did the difficult breathing or breathlessness last?	☐☐<br>(Use 99 if 'Don't know' and 88 if 'Refused to answer')
b) Was the difficulty in breathing continuous, on and off, or only at night?	☐ 1.continuous ☐ 2.on and off<br>☐ 3.only at night ☐ 4.don't know<br>☐ 5.refused to answer
c) Was s/he breathless while lying flat?	☐ 1. Yes ☐ 2. No ☐ 3. don't know<br>☐ 4. refused to answer
A26. Did s/he have chest pain?	☐ 1. Yes ☐ 2. No ☐ 3. don't know<br>☐ 4. refused to answer
**If 'NO', 'Don't know', or 'Refused to answer' skip to question A27**	
a) Was the chest pain severe?	☐ 1. Yes ☐ 2. No ☐ 3. don't know<br>☐ 4. refused to answer
A27. Did he/she have a stiff or painful neck during the illness that led to death?	☐ 1. Yes ☐ 2. No ☐ 3. don't know<br>☐ 4. refused to answer
**If 'NO' , 'Don't know', or 'Refused to answer' skip to question A28**	
a) i. How long before death did s/he have a stiff neck?	☐ 1. Days ☐ 2. Months ☐ 3. don't know<br>☐ 4. refused to answer
**If 'Don't know' or 'Refused to answer', skip to question A28**	
a) ii. How many days/ months before death did s/he have a stiff neck?	☐☐<br>(Use 99 if 'Don't know' and 88 if 'Refused to answer')
A28. Was he/she unconscious during the illness that led to death?	☐ 1. Yes ☐ 2. No ☐ 3. don't know<br>☐ 4. refused to answer
**If 'NO', 'Don't know', or 'Refused to answer' skip to question A29**	
a) i. How long before death did unconsciousness start?	☐ 1. Days ☐ 2. Months ☐ 3. don't know<br>☐ 4. refused to answer
**If 'Don't know' or 'Refused to answer', skip to question A28. b)**	

a) ii. How many days/ months before death did unconsciousness start?	☐☐ (Use 99 if 'Don't know' and 88 if 'Refused to answer')
b) Did the unconsciousness start suddenly, quickly (at most within a single day)?	☐ 1. Yes ☐ 2. No ☐ 3. don't know ☐ 4. refused to answer
A29. Was s/he suddenly unable to talk?	☐ 1. Yes ☐ 2. No ☐ 3. don't know ☐ 4. refused to answer
A30. During the illness that led to death, did he/she have convulsions?	☐ 1. Yes ☐ 2. No ☐ 3. don't know ☐ 4. refused to answer
A31. Did the deceased have any urine problems?	☐ 1. Yes ☐ 2. No ☐ 3. don't know ☐ 4. refused to answer
**If 'NO', 'Don't know', or 'Refused to answer' skip to question A33**	
A32. Did s/he has difficulty passing urine, passed very little or no urine?	☐ 1. Yes ☐ 2. No ☐ 3. don't know ☐ 4. refused to answer
A33. During the illness that led to death, did he/she have any skin rash?	☐ 1. Yes ☐ 2. No ☐ 3. don't know ☐ 4. refused to answer
**If 'NO', 'Don't know', or 'Refused to answer' skip to question A34**	
a) i. How long did the rash last?	☐ 1. Days ☐ 2. Months ☐ 3. doesn't know ☐ 4. refused to answer
**If 'Don't know' or 'Refused to answer', skip to question A34**	
a) ii. How many days/ months did the rash last?	☐☐ (Use 99 if 'Don't know' and 88 if 'Refused to answer')
A34. Did s/he have noticeable weight loss? [hint: limbs (legs, arms) become very thin]	☐ 1. Yes ☐ 2. No ☐ 3. don't know ☐ 4. refused to answer
**If 'NO', 'Don't know', or 'Refused to answer' skip to question A35**	
a) Was s/he severely thinning or wasted? (show photo)	☐ 1. Yes ☐ 2. No ☐ 3. don't know ☐ 4. refused to answer
A35. During the illness that led to death, did he/she have swelling around the ankles?	☐ 1. Yes ☐ 2. No ☐ 3. don't know ☐ 4. refused to answer
A36. Did s/he have yellow discoloration of the eyes?	☐ 1. Yes ☐ 2. No ☐ 3. don't know ☐ 4. refused to answer
A37. Did s/he look pale (thinning/lack of blood) or have pale palms, eyes or nail bed?	☐ 1. Yes ☐ 2. No ☐ 3. don't know ☐ 4. refused to answer
A38. Did s/he have stiffness of the whole body or was unable to open the mouth?	☐ 1. Yes ☐ 2. No ☐ 3. don't know ☐ 4. refused to answer
**If 'NO', '**Don't know', or 'Refused to answer' **skip to question A39**	
a) i. How long did this last?	☐ 1. Days ☐ 2. Months ☐ 3. Years

	☐ 4. don't know ☐ 5. refused to answer
**If 'Don't know' or 'Refused to answer', skip to question A39**	
a) ii. How many days/ months/ years did this last?	☐☐ (Use 99 if 'Don't know' and 88 if 'Refused to answer')
A39. Did s/he have difficulty swallowing?	☐ 1. Yes ☐ 2. No ☐ 3. don't know ☐ 4. refused to answer
**If 'NO', 'Don't know', or 'Refused to answer' continue from question A40 (women only questionnaire) in case the deceased person is a woman. Otherwise for men skip to question A45 (General Questionnaire)**	
a) i. For how long before death did s/he have difficulty swallowing?	☐ 1. Days ☐ 2. Months ☐ 3. Years ☐ 4. don't know ☐ 5. refused to answer
**If 'Don't know' or 'Refused to answer', skip to question A40**	
a) ii. For how many days/ months/ years before death did s/he have difficulty swallowing?	☐☐ (Use 99 if 'Don't know' and 88 if 'Refused to answer')
**Questions A40-A44 are for women only. For men skip to A45**	
A40. Did she have any lump(s) or ulcers (pits) in the breast?	☐ 1. Yes ☐ 2. No ☐ 3. don't know ☐ 4. refused to answer
**If 'NO', '**Don't know', or 'Refused to answer' **skip to question A41**	
a) i. For how long did she have the lump(s)/ulcers?	☐ 1. Days ☐ 2. Months ☐ 3. Years ☐ 4. don't know ☐ 5. refused to answer
**If 'Don't know' or 'Refused to answer', skip to question A41**	
a) ii. For how many days/ months/ years did she have the lump(s)/ulcers?	☐☐ (Use 99 if 'Don't know' and 88 if 'Refused to answer')
A41. Was there any abnormal vaginal bleeding?	☐ 1. Yes ☐ 2. No ☐ 3. don't know ☐ 4. refused to answer
**If 'NO', '**Don't know', or 'Refused to answer' **skip to question A42**	
a) i. How long did the abnormal bleeding last?	☐ 1. Days ☐ 2. Months ☐ 3. don't know ☐ 4. refused to answer
**If 'Don't know' or 'Refused to answer', skip to question A42**	
a) ii. How many days/ months did the abnormal bleeding last?	☐☐ (Use 99 if 'Don't know' and 88 if 'Refused to answer')
A42. Was she pregnant (and not yet in labor) at the time of death?	☐ 1. Yes ☐ 2. No ☐ 3. don't know ☐ 4. refused to answer
**If 'NO', 'Don't know', or 'Refused to answer' skip to question A43**	

a) For how many months was she pregnant?	☐☐ months<br>(Use 99 if 'Don't know' and 88 if 'Refused to answer')
b) Was there vaginal bleeding during the last three months of pregnancy but before labor started?	☐ 1. Yes ☐ 2. No ☐ 3. don't know<br>☐ 4. refused to answer
c) Did she have new abdominal (belly) or back pain during the last three months of pregnancy but before labor started?	☐ 1. Yes ☐ 2. No ☐ 3. don't know<br>☐ 4. refused to answer
d) Did she suffer from convulsions during the last 3 months of the pregnancy?	☐ 1. Yes ☐ 2. No ☐ 3. don't know<br>☐ 4. refused to answer
**After completing question A42. d), skip to question A45**	
A43. Did she have a pregnancy that ended in an abortion or miscarriage within 6 weeks before her death?	☐ 1. Yes ☐ 2. No ☐ 3. don't know<br>☐ 4. refused to answer
**If 'NO', 'Don't know', or 'Refused to answer' skip to question A44**	
a. For how many months was she pregnant?	☐☐ Months<br>(Use 99 if 'Don't know' and 88 if 'Refused to answer')
b. Did she suffer from convulsions while she was pregnant or during or after the abortion/miscarriage?	☐ 1. Yes ☐ 2. No ☐ 3. don't know<br>☐ 4. refused to answer
c. Did she have excessive bleeding during or after the abortion/miscarriage?	☐ 1. Yes ☐ 2. No ☐ 3. don't know<br>☐ 4. refused to answer
**After completing question A43.c, skip to question A45**	
A44. Did she die during labor or in delivery or within 6 weeks after delivering her child ?	☐ 1. Yes ☐ 2. No ☐ 3. don't know<br>☐ 4. refused to answer
**If 'NO', 'Don't know', or 'Refused to answer' skip to question A45**	
a) How many months long was the pregnancy?	☐☐ Months<br>(Use 99 if 'Don't know' and 88 if 'Refused to answer')
b) Was there excessive bleeding during labor or delivery?	☐ 1. Yes ☐ 2. No ☐ 3. don't know<br>☐ 4. refused to answer
c) Did she suffer from convulsions during the last 3 months of pregnancy or during labor or delivery?	☐ 1. Yes ☐ 2. No ☐ 3. don't know<br>☐ 4. refused to answer
d) Did she die after delivering a baby?	☐ 1. Yes ☐ 2. No ☐ 3. don't know<br>☐ 4. refused to answer
**If 'NO', 'Don't know', or 'Refused to answer' skip to subset question no. "h"**	

e) i. How long after delivering the baby did she die?	☐ 1. Days ☐ 2. Weeks ☐ 3. don't know ☐ 4. refused to answer
**If 'Don't know' or 'Refused to answer', skip to question A44. f)**	
e) ii. How many days/ weeks after delivering the baby did; she die?	☐☐ (Use 99 if 'Don't know' and 88 if 'Refused to answer')
f) Was there excessive bleeding after delivery?	☐ 1. Yes ☐ 2. No ☐ 3. don't know ☐ 4. refused to answer
g) Did she suffer from convulsions after delivery?	☐ 1. Yes ☐ 2. No ☐ 3. doesn't know ☐ 4. refused to answer
h) For how many hours was she in labor?	☐☐ hours (Use 99 if 'Don't know' and 88 if 'Refused to answer')
i) Was the delivery normal vaginal, without forceps or vacuum?	☐ 1. Yes ☐ 2. No ☐ 3. don't know ☐ 4. refused to answer
**If, 'YES' skip to question A45**	
j) Was the delivery vaginal, with forceps or vacuum?	☐ 1. Yes ☐ 2. No ☐ 3. don't know ☐ 4. refused to answer
**If 'YES' skip to question A45**	
k) Was the delivery a Caesarean section?	☐ 1. Yes ☐ 2. No ☐ 3. don't know ☐ 4. refused to answer
**General questionnaire (for all deaths):**	
A45. Did he/she suffer from any injury or accident that led to her/his death?	☐ 1. Yes ☐ 2. No ☐ 3. don't know ☐ 4. refused to answer
**If 'NO','Don't know', or 'Refused to answer' skip to question A48**	
A46. What was the injury or accident? Was it:	
**Skip to question A47. i), if any of the below questions (A46.a – A46.j) returns a 'YES' response**	
a) a road traffic crash/injury?	☐ 1. Yes ☐ 2. No ☐ 3. don't know ☐ 4. refused to answer
b) a fall?	☐ 1. Yes ☐ 2. No ☐ 3. don't know ☐ 4. refused to answer
c) drowning?	☐ 1. Yes ☐ 2. No ☐ 3. don't know ☐ 4. refused to answer
d) poisoning?	☐ 1. Yes ☐ 2. No ☐ 3. don't know ☐ 4. refused to answer
e) Any animal bites?	☐ 1. Yes ☐ 2. No ☐ 3. don't know ☐ 4. refused to answer

f) A bite or sting by a venomous animal?	☐ 1. Yes ☐ 2. No ☐ 3. don't know<br>☐ 4. refused to answer
g) A burn?	☐ 1. Yes ☐ 2. No ☐ 3. don't know<br>☐ 4. refused to answer
h) Any other injury?	☐ 1. Yes ☐ 2. No ☐ 3. don't know<br>☐ 4. refused to answer
i) Intentionally inflicted by someone else (homicide, abuse)?	☐ 1. Yes ☐ 2. No ☐ 3. don't know<br>☐ 4. refused to answer
j) Self-inflicted?	☐ 1. Yes ☐ 2. No ☐ 3. don't know<br>☐ 4. refused to answer
A47. i. How long after the injury or accident did s/he die?	☐ 1. hours ☐ 2. days ☐ 3. months<br>☐ 4. Don't know ☐ 5. Refused to answer
**If 'Don't know' or 'Refused to answer', skip to question A48**	
A47. ii. How many hours/ days/ months after the injury or accident did s/he die?	☐☐<br>(Use 99 if 'Don't know' and 88 if 'Refused to answer')
A48. Was there any diagnosis by a health professional of tuberculosis?	☐ 1. Yes ☐ 2. No ☐ 3. don't know<br>☐ 4. refused to answer
A49. Was there any diagnosis by a health professional of high blood pressure?	☐ 1. Yes ☐ 2. No ☐ 3. don't know<br>☐ 4. refused to answer
A50. Was there any diagnosis by a health professional of diabetes?	☐ 1. Yes ☐ 2. No ☐ 3. don't know<br>☐ 4. refused to answer
A51. Did the deceased ever have a positive HIV test?	☐ 1. Yes ☐ 2. No ☐ 3. don't know<br>☐ 4. refused to answer
A52. Was there any diagnosis by a health professional that the deceased had AIDS?	☐ 1. Yes ☐ 2. No ☐ 3. don't know<br>☐ 4. refused to answer
A53. Did the deceased's spouse/partner ever have a positive HIV test?	☐ 1. Yes ☐ 2. No ☐ 3. don't know<br>☐ 4. refused to answer
A54. Was there any diagnosis by a health professional that the deceased's spouse/partner had AIDS?	☐ 1. Yes ☐ 2. No ☐ 3. don't know<br>☐ 4. refused to answer
A55. What did the respondent think that this person died of? (Allow the respondent to tell the illness in his or her own words)	

END OF INTERVIEW.

THANK RESPONDENT FOR HER/HIS PARTICIPATION.

Interviewer: Use the below space to write down your comments and observations about the interview, including:

1. Did you have any trouble locating the deceased's household or identifying the best respondent?

2. Did the respondent understand all/most of the questions?

3. Was the respondent interested in the interview and try to answer the questions accurately?

4. Do you have any other comments?
