# Supplementary material for: Determining causes of death through an abridged verbal autopsy tool: a pilot test conducted among vulnerable communities in Kolkata, India
Source: BMJ Glob Health. 2026 Jul 20;11(7):e022829. doi: 10.1136/bmjgh-2025-022829 (PMC13386064; doi:10.1136/bmjgh-2025-022829)

**Supplementary Figures**

Figure S1. Flow diagram showing participant eligibility and sampling process by age group


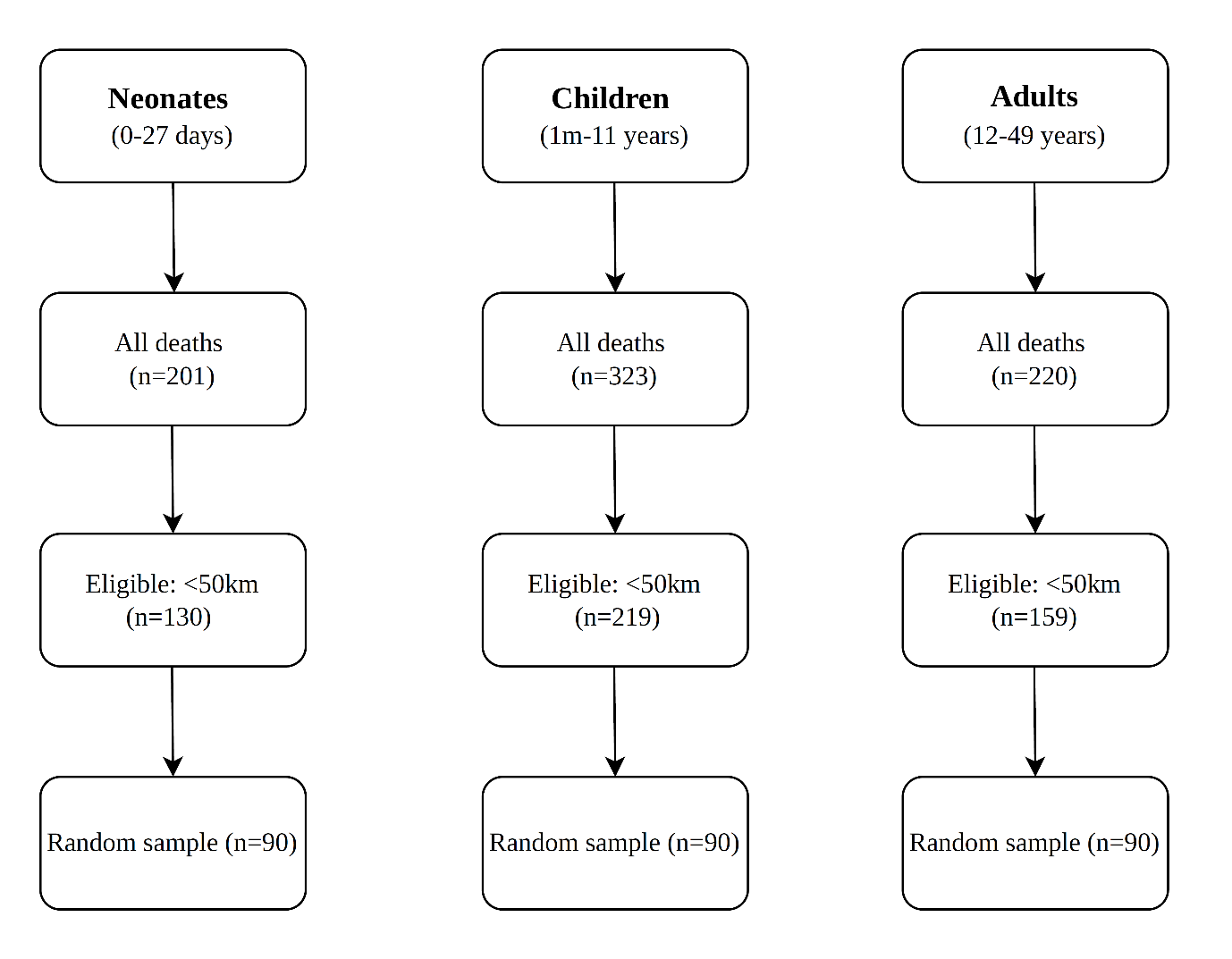

Supplement: online supplemental figure 1 [file bmjgh-11-7-s002.docx]
