# Supplementary material for: Determining causes of death through an abridged verbal autopsy tool: a pilot test conducted among vulnerable communities in Kolkata, India
Source: BMJ Glob Health. 2026 Jul 20;11(7):e022829. doi: 10.1136/bmjgh-2025-022829 (PMC13386064; doi:10.1136/bmjgh-2025-022829)
Supplement: online supplemental file 1 [file bmjgh-11-7-s003.docx]

### BMJ Global Health Author Reflexivity Statement

Adapted from Morton, B., Vercueil, A., Masekela, R., Heinz, E., Reimer, L., Saleh, S., Kalinga, C., Seekles, M., Biccard, B., Chakaya, J., Abimbola, S., Obasi, A. and Oriyo, N. (2022), Consensus statement on measures to promote equitable authorship in the publication of research from international partnerships. Anaesthesia, 77: 264-276. <https://doi.org/10.1111/anae.15597>

| **Study conceptualisation** | |
| --- | --- |
| 1. How does this study address local research and policy priorities? | The study directly responds to India’s national priority of improving mortality surveillance and strengthening public health intelligence under the National Health Mission. It also aligns with WHO’s goal of improving civil registration and vital statistics in low-resource and humanitarian contexts. |
| 1. How were local researchers involved in study design? | The first sentence would be more accurate if it states that the abridged VA tools were developed by the Indian investigators based on the VA algorithms (illness sign/symptom criteria) and corresponding VA questions provided by the JHU investigator (with the adult VA algorithms and questions based on Chandramohan’s paper). |
| **Research management** | |
| 1. How has funding been used to support the local research team(s)? | Funding from the Societies at Risk project (Uppsala University, Sweden) was used entirely for local data collection, fieldwork, and training. No external salaries were paid; all financial resources directly benefited local researchers and institutions. |
| **Data acquisition and analysis** | |
| 1. How are research staff who conducted data collection acknowledged? | All field investigators and study physicians are acknowledged by name as co-authors or in the acknowledgements. |
| 1. How have members of the research partnership been provided with access to study data? | Data are stored securely at ICMR- NIRBI Kolkata, with access provided to all collaborating authors via shared institutional permissions. |
| 1. How were data used to develop analytical skills within the partnership? | The project included structured mentorship in data collection, management, coding, and validation analysis supervised by senior collaborators internationally |
| **Data interpretation** | |
| 1. How have research partners collaborated in interpreting study data? | All authors participated in joint data interpretation meetings and contributed to contextualising findings within the Indian health system  Multiple drafts of the manuscript were produced, with reviewing and editing that stimulated discussion and interpretation of the study data. |
| **Drafting and revising for intellectual content** | |
| 1. How were research partners supported to develop writing skills? | Junior investigators involved in data collection co-drafted the manuscript, with structured mentoring and iterative review by senior Indian and international collaborators. |
| 1. How will research products be shared to address local needs? | Study findings will be shared with the Government of West Bengal, ICMR, and WHO country offices to support integration of the abridged VA tool into local mortality surveillance systems. |
| **Authorship** | |
| 1. How is the leadership, contribution and ownership of this work by LMIC researchers recognised within the authorship? | The study was led by John Hopkins, Uppasala, and ICMR professors/scientists who occupy first, corresponding, and senior author positions. Leadership and intellectual ownership rest primarily with the Indian team. |
| 1. How have early career researchers across the partnership been included within the authorship team? | Early career researchers from ICMR-NIRBI and participating hospitals were included as co-authors to acknowledge their roles in data collection, analysis, and manuscript development. |
| 1. How has gender balance been addressed within the authorship? | The author team includes both male and female researchers in leadership and analytical roles, maintaining gender balance across disciplines and career stages. |
| **Training** | |
| 1. How has the project contributed to training of LMIC researchers? | The project trained field researchers and analysts in cause-of-death assignment, ethical interviewing, and data quality assurance. |
| **Infrastructure** | |
| 1. How has the project contributed to improvements in local infrastructure? | Although no new infrastructure was established, the project enhanced local research processes through training, standardised data collection practices, and improved collaboration between hospitals and research institutions |
| **Governance** | |
| 1. What safeguarding procedures were used to protect local study participants and researchers? | Verbal autopsies were conducted only after written informed consent from bereaved family members, in private and supportive environments. Field staff received training in trauma-sensitive interviewing and confidentiality protocols to protect both respondents and data collectors. |
